# N-Homocysteinylation of HMGB1/2 Promotes Corpus Cavernosum Endothelial Senescence in Erectile Dysfunction

**DOI:** 10.7150/ijbs.119514

**Published:** 2025-10-20

**Authors:** Peng Hu, Sen Fu, Beining Li, Xiaoyu Zhu, Bocheng Tu, Chenglin Han, Jiaxin Wang, Wenchao Xu, Xinqi Liu, Shiqing Zhu, Chengwei Wang, Zhiyao Deng, Yuxuan Deng, Sheng Xin, Jingyu Song, Jihong Liu, Kai Cui

**Affiliations:** 1 Department of Urology, Tongji Hospital, Tongji Medical College, Huazhong University of Science and Technology, Wuhan 430030, China.; 2 Institute of Urology, Tongji Hospital, Tongji Medical College, Huazhong University of Science and Technology, Wuhan 430030, China.; 3 Department of Urology, The First Affiliated Hospital of Shandong First Medical University & Shandong Provincial Qianfoshan Hospital, Jinan, Shandong, China.; 4 Department of Neurosurgery, Tongji Hospital of Tongji Medical College of Huazhong University of Science and Technology, Wuhan 430030, China.; 5 Department of Plastic and Cosmetic Surgery, Tongji Hospital of Tongji Medical College of Huazhong University of Science and Technology, Wuhan 430030, China.

**Keywords:** erectile dysfunction, cellular senescence, N-homocysteinylation, methionyl-tRNA synthetase 1, High mobility group box 1 and 2

## Abstract

Homocysteine (Hcy) is an age-related risk factor for erectile dysfunction (ED), with enhanced vascular toxicity in middle-aged and elderly individuals. However, folate-based Hcy-lowering therapies have shown limited efficacy, necessitating a reevaluation of its age-dependent pathogenic mechanism. Here, we demonstrate that senescent endothelial cells exhibit heightened responsiveness of methionyl-tRNA synthetase 1 (MARS1) to Hcy, promoting the production of homocysteine thiolactone (HTL) and widespread N-homocysteinylation (K-Hcy) of proteins. K-Hcy, rather than acetylation, drives cytoplasmic translocation and extracellular release of high mobility group box proteins 1 and 2 (HMGB1/2), amplifying the senescence-associated secretory phenotype (SASP). Competitive inhibition of MARS1 with N-acetylcysteine (NAC) attenuates endothelial senescence and improves erectile function in middle-aged individuals with hyperhomocysteinemia by reducing HTL, rather than Hcy itself, while synergizing with tadalafil. Collectively, our findings highlight the pivotal role of the age-dependent MARS1-HTL axis in the pathogenesis of homocysteine-induced ED, offering a promising therapeutic strategy for ED in the aging population.

## Introduction

Erectile dysfunction (ED), a prevalent age-related condition, affects over 50% of men aged 40-70 years and nearly 70% of those over 70, significantly impairing quality of life and serving as an early marker for systemic vascular pathologies^1^. Beyond the natural decline in sexual function associated with aging, ED in the elderly is frequently linked to metabolic disorders. In recent years, hyperhomocysteinemia (HHcy) has emerged as a significant risk factor for ED, particularly in aging populations^2^, ^3^. Clinical data indicate that the prevalence of HHcy increases with age, and the detrimental impact of homocysteine (Hcy) on erectile dysfunction (ED) becomes more pronounced as age advances^4^. However, the interaction between Hcy and aging in ED, as well as its underlying mechanisms, remains unknown.

Hcy is a key contributor to vascular aging, promoting endothelial dysfunction through oxidative stress, inflammation, and nitric oxide (NO) depletion^5^, ^6^. Two meta-analyses have reported that patients with ED exhibit elevated serum Hcy levels, an association primarily derived from observational case-control studies^7^-^9^. Whether folic acid, a common therapy for HHcy, can directly improve ED by lowering circulating Hcy remains controversial^10^, ^11^. Given the central role of hemodynamics in erectile physiology, it is plausible to consider the pathological effects of HHcy documented in other cardiovascular diseases. However, several large RCTs have demonstrated that Hcy-lowering interventions do not reduce the risk of major cardiovascular events^12^, ^13^. Collectively, these inconsistent clinical findings regarding Hcy and Hcy-lowering therapy in ED suggest that a deeper understanding of Hcy metabolism is warranted. In this context, Hcy serves as a substrate for methionyl-tRNA synthetase (MARS), which catalyzes its conversion into homocysteine thiolactone (HTL), a highly reactive metabolite with enhanced cytotoxicity^14^. HTL covalently modifies specific lysine residues on proteins, a process known as N-homocysteinylation (K-Hcy), leading to alterations in protein structure and function^15^. Recent studies on neurodegenerative disorders, including Alzheimer's and Parkinson's diseases, suggest that elevated protein K-Hcy serves as a key pathological mechanism underlying brain senescence^16^-^18^. Accordingly, further research is needed to explore the potential central role of protein K-Hcy in vascular aging associated with ED.

The high-mobility group box 1 (HMGB1) and 2 (HMGB2) proteins, members of the non-histone DNA-binding HMG protein family, are highly conserved and abundant in mammalian nuclei^19^. HMGB1/2 play significant roles in regulating fundamental nuclear processes, including transcription, replication, recombination, and DNA repair^19^. Emerging evidence suggests that nuclear depletion of HMGB1/2 triggers the cellular senescent program through mediating genomic reorganization^20^, ^21^. During cellular senescence, HMGB1 translocates from the nucleus to the cytoplasm. Its relocalization and secretion serve as a paracrine signal, further regulating the senescence-associated secretory phenotype (SASP) in surrounding cells^20^. The active translocation and secretion of HMGB1 are extensively regulated by post-translational modifications (PTMs), including acetylation, phosphorylation, ADP-ribosylation, methylation, glycosylation, and oxidation^22^. Therefore, we hypothesize that HTL enhances K-Hcy of HMGB1/2 to bring about the onset of endothelial senescence and ED.

Aging is intricately linked to metabolic alterations and changes in secretory protein profiles, which collectively influence cellular homeostasis and tissue function^23^. In this study, we found that Hcy promotes endothelial senescence and the development of ED in middle-aged and elderly individuals, with the interaction between Hcy and age primarily mediated by the MARS1-HTL pathway. Furthermore, we elucidated the molecular mechanism by which K-Hcy modification of HMGB1/2 drives endothelial senescence.

## Materials and Methods

### Study participants

The retrospective clinical case-control study included patients diagnosed with erectile dysfunction (ED) and confirmed non-ED control subjects, all recruited from the Urology Department of Tongji Hospital between January 2021 and November 2024. Subjects with a known history of diabetes, coronary artery disease, neurological disorders, pelvic trauma, major psychiatric disorders, thyroid disease, end-stage renal disease, or vitamin deficiencies were excluded from the study.

Data collected included body mass index (BMI), homocysteine (Hcy), triglycerides (TG), total cholesterol (TC), low-density lipoprotein cholesterol (LDL-C), high-density lipoprotein cholesterol (HDL-C), fasting blood glucose (Glu), HbA1c, and smoking history. Participants were stratified by age and matched 1:1 using propensity score matching to adjust for potential confounders, resulting in a final dataset of 240 participants per group for subsequent statistical analysis.

For corpus cavernosum (CC) tissue samples, all donors provided written informed consent after being fully informed of the study's goals and characteristics. Two normal tissue samples were obtained from the tumor margin of penile carcinoma resection in patients who reported good stimulated erections and early morning erections. Four ED cases were obtained from artificial cavernous body implantation samples.

All human study protocols were reviewed and approved by the Human Assurance Committee of Tongji Hospital, Tongji Medical College, Huazhong University of Science and Technology (Approval No. TJ-IRB202412207). Written informed consent was obtained from all patients and control subjects prior to their participation in the study.

### Animal models

All experimental procedures involving animals were approved by the Academic Administration Committee of Tongji Hospital, Tongji Medical College, Huazhong University of Science and Technology (Ethical Approval No. TJH-202204011) and conducted in accordance with the National Institutes of Health Guidelines for the Care and Use of Laboratory Animals. Male Sprague-Dawley (SD) rats were purchased from Beijing HFK Bioscience Co., Ltd., housed under specific pathogen-free (SPF) conditions, and provided food and water at a 12-h light:dark cycle.

SD rats were randomly assigned to the control and experimental groups (n = 10 per group). The young cohort (initially aged 2 months) and mid-old cohort (initially aged 12 months) were established as distinct aging models. Hyperhomocysteinemia (HHcy) was induced by ad libitum administration of L-methionine (Met, 10 g/L dissolved in drinking water) for 6 months^24^, ^25^. Age-matched controls received standard drinking water. Rescue therapies were initiated during the final 2 months of methionine exposure to evaluate reversal potential. The tadalafil (TAD) group received daily oral gavage of 2 mg/kg tadalafil solubilized in a vehicle containing 0.5% carboxymethylcellulose sodium (CMC-Na) and 5% polyethylene glycol 300 (PEG300). The N-acetylcysteine (NAC) group was administered 300 mg/kg NAC dissolved in normal saline via identical gavage protocol. For the combination therapy (T&N) group, both agents were co-delivered at the aforementioned doses using their respective vehicles to assess potential synergistic effects.

### Cell culture and treatments

Primary rat corpus cavernosum endothelial cells (CCECs) were isolated from 6-week-old male Sprague-Dawley rats using enzymatic digestion combined with mechanical extrusion, as previously optimized^26^. Briefly, dissected cavernosal tissues were minced into 1-3 mm³ fragments and digested with collagenase Type II (0.25%, 40 min) at 37 °C, followed by mechanical disruption using the rubber tip of a syringe to enhance dissociation. Cell suspensions were filtered (100 μm), centrifuged (600 ×g, 5 min), and cultured in Endothelial Cell Medium (ECM) supplemented with 5% FBS. Primary CCECs (P1) were purified via fixed-point digestion: cobblestone-shaped regions were selectively treated with 0.25% trypsin (1-2 min), and further purification was performed using Endothelial Cell Isolation Kit (130-109-679; Miltenyi). Cultures were maintained at 37 °C/5% CO₂, with medium changes every 2-3 days. CCECs from passage 1 to passage 9 were employed to investigate the dynamic changes in Mars1 and K-Hcy expression during consecutive subcultures.

Human cardiac microvascular endothelial cells (HCMECs) and HEK293T were obtained from Warner Bio and cultured for transfection experiments and mechanistic studies. HEK293F cells were used for protein purification. HCMECs were cultured in ECM supplemented with 5% FBS, while HEK293F cells were cultured in SMM 293-TII Expression Medium (SinoBiological Inc., M293TII). Cells were grown at 37 °C/5% CO₂, and their mycoplasma status was regularly checked to ensure the absence of contamination. Hcy, HTL, and N-acetyl-cysteine (NAC) were purchased from Sigma-Aldrich. For the induction of cellular senescence, Hcy (200 µM) or HTL (100 µM) was applied for 4 days. NAC (2 mM) treatment was administered. For the K-Hcy modification proteomic analysis, 1 mM Hcy or HTL was applied for 24 hours. HCMECs at passages 10-15 were used for MARS1 knockdown experiments, while those at passages 3-8 were applied for MARS1 and/or HMGB1/2 overexpression studies. For comparative analyses of bidirectional MARS1 interventions, cells at passages 6-10 were employed and defined as the “mid-old” state.

### Antibodies

Commercial antibodies were purchased from the suppliers listed in Appendix Table S1. Custom Anti-K-Hcy antibody was produced following established protocols^27^, ^28^. Briefly, 10 mg of chicken egg albumin (OVA) was homocysteinylated by incubation with 60 mg HTL in 10 mL of 0.1 M Na₂CO₃ (pH 8.0) containing 200 μl pyridine at room temperature overnight. The reaction was then supplemented with 15 mM iodoacetamide and incubated in the dark at room temperature for 1 hour. The mixture was desalted by passing it through a G-25 column in an AKTA-FPLC system and dried in a desiccator to obtain the homocysteine-modified antigen, which was subsequently sent to Abmart (Abmart Shanghai Co., Ltd) for antibody production.

### Measurement of erectile function

Erectile function was assessed *in vivo* by recording intracavernosal pressure (ICP) in response to cavernous nerve stimulation in anesthetized male rats^29^. A 25-gauge heparinized needle connected to a pressure transducer (BIOPAC MP160, USA) was inserted into the corpus cavernosum, while systemic blood pressure was monitored via a carotid artery catheter. Electrical stimulation (15 Hz, 5.0 V, 60 s) was applied using a bipolar platinum electrode. The maximum ICP and the area under the curve (total ICP) were both normalized to mean arterial pressure (MAP) and analyzed. After recordings, rats were euthanized, and corpus cavernosum tissue was collected for further study.

### Blood flow analysis

Rats were anesthetized with isoflurane (induction at 4-5%, maintenance at 1.5-2%) and placed in a supine position on a heating pad (37 °C). For measurements of penile body blood flow^30^, the penis was exposed by gently pressing the rat's abdomen to allow the glans penis to surface, followed by immobilization of the organ by gently clamping the male urogenital mating protuberance. Basal blood flow was recorded 2-5 minutes after organ positioning with an automatic focal distance using the RFLSI Ⅲ blood perfusion imager (RWD Life Science, China). The system employs laser speckle contrast analysis (LASCA) with a 785 nm laser and a CCD camera (2048x2048 pixels) for image capture (0.25 Hz). An erection was induced by injecting 5 μL of sodium nitroprusside (NO donor, 25mM) into the glans penis, and stable blood flow was recorded 5-10 minutes post-injection. The percentage change in blood flow was calculated as (Post-Injection Flow-Basal Flow)/Basal Flow×100%.

### Detection of homocysteine and HTL

Hcy quantification was performed using a Homocysteine-EIA Kit (Axis-Shield) with matrix-specific pretreatments: intracellular samples were lysed and centrifuged (10,000 ×g, 4 °C, 10 min); EDTA-treated plasma samples were clarified by centrifugation (1,000 ×g, 4 °C, 10 min); penile tissues were homogenized in ice-cold PBS and centrifuged (10,000 ×g, 4 °C, 10 min). For HTL measurement, cells or homogenized penile tissues were denatured with pre-chilled 80% methanol, followed by centrifugation (10,000 ×g, 4 °C, 10 min). The supernatants were collected, vacuum-dried, re-dissolved in ddH2O, and subjected to ultrafiltration using a polyvinylidene fluoride membrane (Millex-GV, Millipore). The metabolites were extracted, and HTL was analyzed by LC-MS as previously described^18^.

### Single cell RNA-sequencing analysis

Freshly dissected rat corpus cavernosum tissues were rinsed and vigorously agitated in ice-cold PBS to remove residual blood cells, followed by mincing and enzymatic digestion with 2.5 mg/ml collagenase type I, 4 mg/ml collagenase type IV, 0.1 mg/ml neutral protease, and 2 mg/ml DNase I at 37°C for 40 min^31^. The dissociated cells were filtered through a 40-μm strainer, and single-cell suspensions with viability > 90% were processed on the DNBSEQ T7 platform at the HaploX Biotechnology (JiangXi, China). Barcoding and cDNA synthesis were performed using the DNBelab C-Series High-throughput Single-cell RNA Library Preparation Set V3.0 (TaiM 4, MGI, #940-001819-00) according to the manufacturer's instructions.

Sequencing data were further processed using DNBC4tools for alignment (Rattus norvegicus, Ensembl release-110) and UMI counting. Low-quality cells were filtered according to the following threshold parameters: the total number of expressed genes, 200-3500; total UMI count, > 1000 and below the 97th percentile; and proportion of mitochondrial genes expressed, < 20%. Normalization, batch correction (RunHarmony), and clustering were performed using the Seurat (v4.3.0) standard workflow, and cell types were annotated based on canonical markers^32^. Differentially expressed genes (DEGs) were identified using the Seurat function FindAllMarkers (test.use = "wilcox", min.pct = 0.1, logfc.threshold = 0.25) based on normalized UMI counts. Monocle was used for pseudotime trajectory analysis, and CellChat was employed to analyze intercellular communications^33^, ^34^.

### SA-β-Gal staining

SA-β-Gal staining was performed using the Senescence-Associated β-Galactosidase Stain Kit (G1580, Solarbio) following the manufacturer's instructions. For tissue staining, freshly dissected rat corpus cavernosum tissues were embedded in OCT compound, cryosectioned at 10 μm, and fixed with the β-Gal fixative solution provided in the kit at room temperature for 30 min. For cell staining, cultured cells were seeded in 12-well plates, washed with PBS, and fixed under the same conditions. After fixation, both tissue sections and cells were incubated with SA-β-Gal staining solution at 37 °C overnight in a CO₂-free environment. Stained samples were examined using a bright-field microscope, and blue precipitates indicated positive staining.

### Immunofluorescence

The penile tissues were carefully dissected and fixed in 4% paraformaldehyde overnight at 4 ℃, followed by overnight dehydration in 30% sucrose. Frozen sections (5 μm) were prepared and for antigen retrieval, sections were incubated in 0.01 M sodium citrate buffer in a microwave oven. Sections were permeabilized with 0.1% Triton X-100 for 15 minutes and blocked with 4% bovine serum albumin (BSA) for 1 hour at room temperature. Primary antibodies targeting specific markers (Table S1) were applied overnight at 4°C. After washing, fluorescently labeled secondary antibodies were incubated for 1 hour at room temperature. Nuclei were counterstained with DAPI, and slides were mounted with antifade medium. Images were acquired using a fluorescence microscope (Zeiss Axio Imager M2), and fluorescence intensity was quantified using ImageJ software.

### Western blotting

Tissues or cells were lysed in RIPA buffer supplemented with protease and phosphatase inhibitors. Protein concentrations were determined using a BCA assay, and equal amounts of protein were separated by SDS-PAGE and transferred to PVDF membranes. Membranes were blocked with protein-free rapid blocking buffer (Boster Biological Technology, AR0041) for 15 minutes at room temperature, followed by incubation with primary antibodies overnight at 4 °C (Table S1). After washing, membranes were incubated with HRP-conjugated secondary antibodies for 1 hour at room temperature. Protein bands were visualized using enhanced chemiluminescence (ECL) and quantified using ImageLab software. β-actin or Vinculin was used as a loading control.

### Plasmid constructs, lentivirus packaging, and transfection

Plasmid construction was performed by restriction enzyme digestion and ligation or Gibson assembly methods. The target genes were amplified using specific primers and cloned into the desired vectors, including pcDNA3.1, pLenti-EnCMV, pLKO.1, pBiFC, and pGEX-4T-1, to generate fusion constructs. Detailed plasmid information is provided in Appendix Table S2.

Lentivirus packaging was carried out by co-transfecting HEK293T cells with pLenti-EnCMV-based plasmids and packaging vectors (pMD2.G and psPAX2) using Lipofectamine 3000 (Invitrogen, L3000015). After 48 hours of incubation, the viral supernatant was collected, filtered, and concentrated by ultracentrifugation.

For stable transfection, HCMECs were transduced with lentivirus at a multiplicity of infection (MOI) of 10 and selected with puromycin. For transient transfection, plasmids were introduced into cells using Lipofectamine 3000 according to the manufacturer's instructions.

### Identification of K-Hcy substrates

#### Antibody-based K-Hcy proteomics

Following Hcy treatment, HCMEC cells were lysed in 1% sodium deoxycholate/50 mM Tris-HCl (pH 8.5) with protease inhibitors. Lysates were sonicated (3 × 10 s pulses, 30% amplitude), centrifuged (12,000 × g, 10 min, 4 °C), and the supernatants collected. Protein concentration was determined by Bradford assay. 5 mg of extracted proteins were reduced and alkylated with TCEP and IAA at 37 °C for 1 h. The sample was diluted 4-fold with 25 mM ammonium bicarbonate (ABC) buffer, followed by overnight digestion with trypsin (1:50) at 37 °C. The digestion was terminated by adding 0.1% FA. For peptide clean-up, the sample was loaded onto a C18 column, washed with 0.1% FA and pure water, and eluted with 70% ACN. The eluates were combined, lyophilized, and stored at -80 °C until further analysis.

Immunoprecipitation (IP) was performed using pan-K-Hcy antibody conjugated to Protein A/G beads. Briefly, the peptides were incubated with the antibody-bead complex for 4 hours at room temperature, followed by washing with IP buffer to remove non-specifically bound peptides. The eluted peptides were resuspended in 0.1% FA and directly analyzed by LC-MS/MS at SpecAlly Life Technology (SpecAlly Life Technology Co., Ltd.). Briefly, samples were analyzed on a timsTOF Pro mass spectrometer (Bruker Daltonics) coupled with an UltiMate 3000 RSLCnano system (Thermo). Peptides were separated on a C18 Trap column and a C18 analytical column (IonOpticks) with a 300 nL/min flow rate using mobile phases A (0.1% FA in water) and B (0.1% FA in ACN). MS data were acquired in PASEF mode with a capillary voltage of 1500 V, scanning from 100 to 1700 m/z. Ion mobility was scanned from 0.75 to 1.4 Vs/cm², and a 1.88 s acquisition cycle included one full MS scan and 10 PASEF MS/MS scans. Singly charged precursors above an intensity threshold of 1000 were fragmented. Data were processed with MaxQuant (2.0.1.0) and searched against the human protein database (Uniprot, 20230619) with a 1% FDR at both peptide and protein levels. Variable modifications included Carbamidomethyl (C), Oxidation (M), Acetylation (Protein N-terminal), and N-homocysteinylation (K, +174.04600 Da), and enzyme specificity was set to Trypsin/P with a maximum of 2 missed cleavages.

#### Chemoselective labeling-based K-Hcy differential proteomics

 HCMEC cells were cultured in ECM with or without Hcy (200 μM) for 24 hours. After harvesting by scraping, proteins were extracted by lysis in RIPA buffer (Solarbio, R0010) with protease inhibitors, followed by sonication and centrifugation. For chemoselective labeling^35^, 1 mg of total proteins per sample were diluted in PBS (0.2 mM EDTA, pH 9). The reaction solution was labeled with Biotin-azide (200 μM, MedChemExpress, HY-129832). Freshly prepared hemin (50 μM), β-Mercaptoethanol (100 mM), and SDS (0.4%) were added, and the mixture was heated at 75°C for 10 minutes, followed by acetone precipitation. The protein pellets were washed twice with 70% acetone and resuspended in SDS/PBS (1.2%) for sonication. After centrifugation (20,000 ×g, 3 min), proteins were diluted in PBS containing 0.2% SDS and enriched with streptavidin magnetic beads (MedChemExpress, HY-K0208) for 3 hours at room temperature with rotation. The beads were then washed sequentially with PBS and water. Proteins were eluted by biotin competition, followed by reduction, alkylation, and trypsin digestion. The resulting peptides were sent to SpecAlly Life Technology for quantitative LC-MS/MS analysis.

#### HMGB1/2 K-Hcy site identification

 For detecting K-Hcy modification sites on HMGB1/2, HCMEC cells were transfected with HMGB1/2-Flag vectors and treated with HTL for 24 hours. Cells were lysed in 0.5% NP-40 buffer supplemented with 5 mM iodoacetamide and protease inhibitors. The lysates were immunoprecipitated using anti-FLAG magnetic beads (MedChemExpress, HY-K0207) for 3 hours at room temperature. Elution-tryptic digestion was performed at 37 °C overnight. Peptides were stored at -80 °C for subsequent LC-MS/MS analysis.

### CUT&Tag assay

CUT&Tag was performed using the Vazyme TD904 kit according to the manufacturer's protocol^36^. Briefly, cells were harvested and immobilized on concanavalin A-coated magnetic beads, followed by permeabilization with digitonin buffer. Cells were then incubated with primary antibodies (Table S1) at 4 °C overnight, followed by secondary antibody binding. Tn5 transposase was then introduced for targeted chromatin cleavage and tagging. After tagmentation, DNA was extracted, PCR-amplified, and purified for sequencing. Libraries were sequenced on an Illumina platform, and data were processed using CUT&Tag-specific bioinformatics pipelines for peak calling and downstream analysis.

### Cytoplasmic and nuclear protein isolation

Nuclear and cytoplasmic proteins were extracted using the Nuclear-Cytosol Extraction Kit (Applygen Technologies Inc., P1200) according to the manufacturer's instructions. Briefly, cells were harvested and washed with PBS, followed by resuspension in Cytosol Extraction Buffer containing protease inhibitors. After incubation on ice for 15 minutes, cells were homogenized and centrifuged at 12,000 × g for 5 minutes at 4 °C. The supernatant (cytoplasmic fraction) was collected, and the pellet was resuspended in Nuclear Extraction Buffer. After vigorous vortexing and centrifugation at 12,000×g for 10 minutes at 4 °C, the supernatant (nuclear fraction) was collected. Protein concentrations were determined using a BCA assay, and samples were stored at -80 °C until further analysis.

### Molecular docking

Molecular docking was performed using AlphaFold3 to predict the three-dimensional structures and interaction complex of the target proteins^37^. The highest-scoring protein-protein complex (Model_0) was selected for further analysis using PyMOL to visualize intermolecular interactions, including hydrogen bonds, salt bridges, and hydrophobic contacts.

### RNA isolation and RT-qPCR

Total RNA was extracted from tissues or cells using TRIzol reagent (Accurate Biology, AG21024) according to the manufacturer's protocol. RNA purity and concentration were assessed using a NanoDrop spectrophotometer (Thermo Fisher). cDNA was synthesized from 1 μg of total RNA using a HiScript III RT SuperMix kit (Vazyme, R323-01). Quantitative PCR was performed using SYBR Green (Vazyme) on a QuantStudio™ 3 system (Applied Biosystems). Relative gene expression was calculated using the 2^-ΔΔCt^ method, with β-actin as the internal control. Primer sequences are listed in Appendix Table S3.

### Protein purification

For prokaryotic protein expression, pGEX-4T-1-HMGB1/2 plasmids fused with a GST tag were transformed into Escherichia coli BL21(DE3) cells. Protein expression was induced by adding 0.1 mM IPTG at 18 °C for 16 hours. The cells were harvested and lysed by sonication in lysis buffer (PBS containing 1% Triton X-100, 10% glycerol, 1 mM EDTA, 1 mM DTT, and protease inhibitors). The lysate was cleared by centrifugation, and the GST-fusion proteins were purified by incubating with Glutathione Agarose Resin (Beyotime, P2251) at 4 °C. After washing, proteins were eluted with 10 mM reduced glutathione in PBS at room temperature.

For eukaryotic protein expression, pcDNA3.1-based plasmids encoding the target genes were transiently transfected into HEK293F cells using PEI reagent, following the manufacturer's instructions. After 72 hours of incubation in serum-free medium, the supernatant was collected for purification. Flag-tagged proteins were captured using Anti-Flag Affinity Gel (Beyotime, P2271), while V5-tagged proteins were purified with Anti-V5 Affinity Gel (Beyotime, P2289). The bound proteins were eluted with their respective peptides, concentrated using 10 kDa cutoff membranes, and stored at -80 °C for future use.

### TurboID proximity labeling

To characterize the differential protein interactions of K-Hcy modified and unmodified extracellular HMGB1/2 in HCMEC, FLAG-TurboID (non-fusion protein) and FLAG-HMGB1/2-TurboID fusion proteins were generated^38^. A signal peptide was added to the N-terminus to ensure proper secretion, and the constructs were expressed in 293F cells. The secreted proteins were purified using anti-FLAG affinity chromatography. For *in vitro* K-Hcy modification, purified proteins were incubated with 1 mM HTL at room temperature for 12 hours.

HCMEC were treated with 0.25 μM TurboID-HMGB1/2 (K-Hcy modified or unmodified), 1 mM ATP, 5 mM MgCl₂, and 500 μM biotin for 1 hour at 37 °C to enable proximity-dependent biotinylation. To stop the reaction, cells were transferred to ice and washed five times with PBS. Cells were then lysed, and biotinylated proteins were enriched using streptavidin magnetic beads (MedChemExpress, HY-K0208). To control for background biotinylation, cells treated with TurboID-FLAG were processed in parallel. The enriched proteins were analyzed by SDS-PAGE followed by silver staining or Western blotting. For proteomic identification, the biotinylated proteins were trypsin-digested and subjected to LC-MS/MS analysis. Data were processed using proteomics software (MaxQuant), and the background signals from TurboID-FLAG alone were subtracted to identify specific interactions of HMGB1/2. Candidate interactions were further validated by GST pull-down and functional assays.

### GST pull-down

For GST pull-down assays, purified GST or GST-fusion HMGB1/2 were immobilized on the glutathione resin at 4°C for 2 hours, followed by incubation with purified SLIT2, SLIT2-N, or SLIT2-C proteins at 4°C for 4 hours. After extensive washing with PBS containing 0.1% Triton X-100, bound proteins were eluted using SDS-PAGE loading buffer and analyzed directly by SDS-PAGE and immunoblotting to detect interactions.

### Bimolecular fluorescence complementation (BiFC)

HEK293T cells were plated on glass-bottom dishes and transfected with VN155(I152L)-SLIT2 and HMGB1/2-VC155 plasmids^39^. After 24 hours, cells were treated with or without HTL for an additional 48 hours. In a separate experiment, cells were co-transfected with VN155(I152L)-SLIT2 and VC155-ROBO4, followed by induction with K-Hcy-modified HMGB1/2 recombinant proteins for 24 hours. After treatment, cells were incubated with Hoechst 33342 (Beyotime, C1028) for nuclear staining and CM-Tracker (Beyotime, C1036) for plasma membrane staining. Fluorescence complementation was observed using a Zeiss LSM900 Airyscan confocal microscope.

### Statistical analysis

Data analysis was performed using R v4.3.1 and GraphPad Prism v10.0. Case-control associations between plasma Hcy levels and ED were evaluated via conditional logistic regression adjusted for potential confounding factors such as age, BMI, smoking status, HbA1c, and LDL-C levels, with results expressed as odds ratios (ORs, 95% CIs). Spearman's correlation coefficient was used to quantify the correlation between Hcy levels and IIEF-5 scores. Receiver operating characteristic (ROC) curves and clinical decision curve analysis (DCA) were used to evaluate the impact of Hcy as a risk factor for ED. The interaction term between age and Hcy was incorporated into the conditional logistic regression model, and its nonlinear component was analyzed using restricted cubic spline (RCS) regression. Normality was assessed via Shapiro-Wilk test. Normally distributed data (mean ± SEM) were analyzed by unpaired two-tailed Student's t-test (two groups) or one-way ANOVA with Tukey's post hoc (≥ 3 groups), while non-normally distributed data (median [IQR]) were analyzed using Mann-Whitney U or Kruskal-Wallis/Dunn's tests. Significance: *p < 0.05, **p < 0.01, ***p < 0.001.

## Results

### Plasma Hcy levels are associated with ED risk in middle-aged and elderly populations

**Figure 1:**
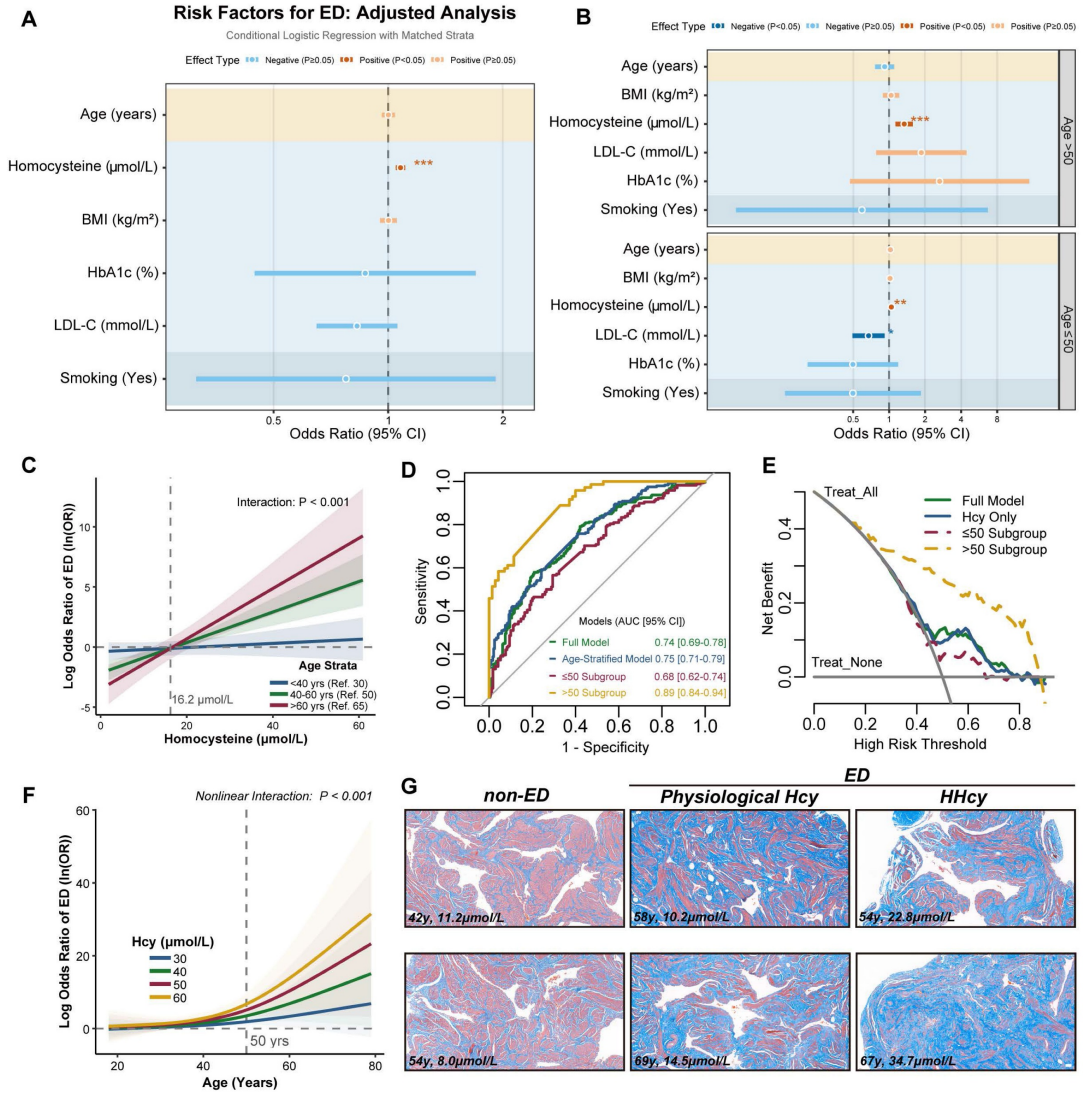
** Plasma Hcy levels are associated with ED risk in middle-aged and elderly populations. A-B.** Odds ratios (OR) of ED risk factors from conditional multivariable logistic regression. Red/blue dots denote positive/negative associations (95% CI). **C.** Predicted log_e_ OR of ED risk from multivariable conditional logistic regression with Hcy-age interaction terms. Curves depict Hcy-associated ED risk across age strata: <40 years (reference=30), 40-60 years (reference=50), and >60 years (reference=65), with shaded 95% confidence bands. **D.** ROC curves evaluating predictive performance of models with age stratification. **E.** Decision curve analysis quantifying net benefit of predictive models with age stratification. **F.** Predicted ED risk curves derived from restricted cubic spline (RCS) regression with Hcy-age interaction terms. **G.** Representative Masson's trichrome-stained histopathological sections of human corpora cavernosa: non-ED control, physiological Hcy, and hyperhomocysteinemia related ED. Collagen fibers (blue), smooth muscle (red). Scale bars: 50 μm.

To investigate the potential association between Hcy levels and the risk of ED, we conducted a retrospective case-control study involving 480 participants (240 ED cases and 240 covariate-matched controls). Baseline characteristics revealed that ED patients exhibited significantly higher median Hcy levels compared to controls (14.4 [11.5-23.0] vs. 12.7 [10.0-15.8] μmol/L, *p* < 0.001; Table 1). Notably, this difference was further amplified in individuals aged > 60 years (23.4 [20.9-29.6] vs. 13.3 [11.1-16.4] μmol/L; *p* < 0.001; Table 2). Multivariable conditional logistic regression identified Hcy as an independent risk factor for ED (OR= 1.077, 95% CI: 1.046-1.109, p < 0.001; Figure 1A-B). Notably, age-stratified analysis revealed a heightened association in individuals > 50 years, where Hcy exhibited a stronger effect size (OR = 1.337, 95% CI: 1.131-1.581, p < 0.001).

Next, we incorporated Hcy-age interaction terms into multivariable conditional logistic regression, revealing a pronounced age-dependent escalation in Hcy-associated ED risk. Individuals aged > 60 years exhibited a significantly steeper risk slope compared to younger counterparts (Figure 1C). AUC analysis revealed superior diagnostic performance of the model in the > 50-year subgroup (AUC = 0.89, 95% CI: 0.84-0.94; Figure 1D). Decision curve analysis confirmed the clinical utility of the model, demonstrating a better net benefit at risk thresholds as low as 20% in the > 50-year subgroup (Figure 1E). These findings suggest that early intervention in middle-aged HHcy patients provides substantial clinical benefits. We further employed restricted cubic spline (RCS) regression, which demonstrated a threshold effect: Hcy levels in individuals aged > 50 years drove a steep increase in ED risk (Figure 1F). Masson's trichrome staining of cavernosal tissues demonstrated a significant change in the collagen-to-smooth muscle ratio in HHcy-associated ED cases compared to non-ED and naturally aging ED patients, providing structural evidence for Hcy-mediated corpora cavernosa vascular sinus remodeling (Figure 1G). These results suggest that plasma Hcy levels are associated with ED risk in middle-aged and elderly populations, with a potential interaction between age and Hcy.

### HHcy induces ED in middle-aged rats via accelerated endothelial senescence

**Figure 2:**
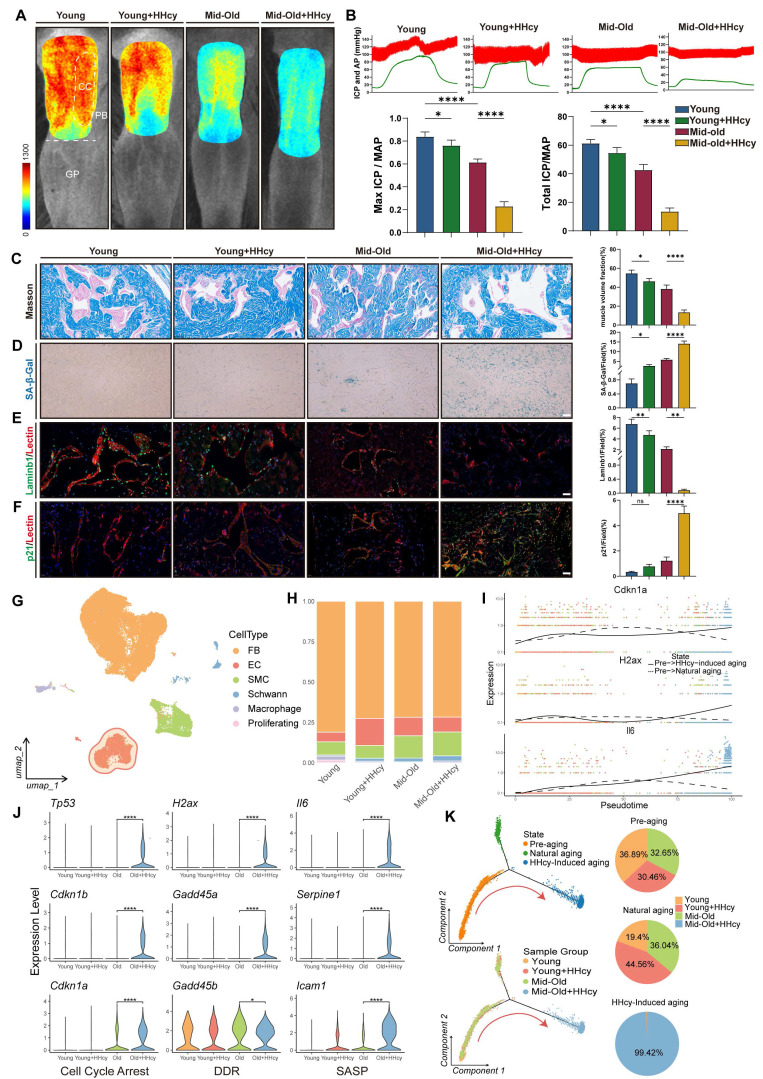
** HHcy induces ED in middle-aged rats via accelerated endothelial senescence. A.** Representative laser speckle contrast imaging of sodium nitroprusside (NO donor)-induced rat penile blood flow across four experimental groups (n=3): Young (control), Young+HHcy, Mid-old (natural aging), and Mid-old+HHcy. Color gradients (blue-to-red) indicate relative perfusion levels in anatomical subregions: corpus cavernosum (CC, erectile tissue), penile body (PB), and glans penis (GP). **B.** Representative curves of electrostimulation-induced intracavernosal pressure dynamics and hemodynamic quantification (n=5 per each group). AP: arterial pressure; ICP: intracavernosal pressure; MAP: mean arterial pressure; Total ICP: area under the ICP curve. **C.** Representative images and semi-quantification of Masson's trichrome-stained rat corpus cavernosum sections (n=4). Scale bars: 50 μm. **D.** Representative images and semi-quantification of SA-β-gal activity in rat corpus cavernosum sections (n=4). Scale bars: 50 μm. **E-F.** Representative immunofluorescence co-staining images and semi-quantification in different groups: **E.** Laminb1 (green) and Lectin (endothelium, red). **F.** p21 (green) and Lectin. **G.** UMAP projection plot of the combined single-cell transcriptomes across all experimental groups. FB (Fibroblast), EC (Endothelial cell), SMC (Smooth muscle cell). **H.** Stacked bar plots of single-cell composition across experimental groups. **I.** Pseudotemporal trajectory analysis of senescence-associated genes *Cdkn1a*, *H2ax*, and *Il6* in EC subpopulations. Solid lines represent HHcy-accelerated aging, dashed lines denote natural aging. **J.** Violin plots of senescence-associated gene expression in EC subpopulations across groups. **K.** EC state transition dynamics revealed by Monocle2 pseudotime analysis. Left: Pseudotemporal trajectory of ECs showing bifurcation at Branch Point 1 (senescence initiation). Red arrows indicate HHcy-accelerated transitions from pre-aging (State 1) to senescence state (State 3). Right: Pie charts quantify the proportion of ECs in three pseudotime-defined states.

To investigate whether elevated Hcy levels causally contribute to ED in middle-aged populations, we utilized rat models and employed various in vivo methods to assess vascular and cellular changes. We induced a chronic moderate HHcy rat model by supplementing drinking water with 1% methionine, a widely used approach to study the pathological role of HHcy in cardiovascular diseases. Laser speckle contrast imaging revealed that HHcy significantly impaired erectile penile blood flow in the Mid-old+HHcy group compared to young and naturally aging groups (Figure 2A). This was further supported by intracavernosal pressure (ICP) measurements, which showed a significant reduction in ICP, alongside a lower area under the ICP curve in the Mid-old+HHcy group, indicating diminished erectile function (Figure 2B). Masson's trichrome staining demonstrated an increased collagen-to-smooth muscle ratio in the Mid-old+HHcy group, indicative of vascular remodeling and fibrosis (Figure 2C). Additionally, senescence-associated β-galactosidase (SA-β-gal) activity was elevated in the Mid-old+HHcy group, suggesting accelerated senescence (Figure 2D). Immunofluorescence staining revealed significant endothelial dysfunction and senescence in the HHcy-exposed rats, particularly in the Mid-old+HHcy group, as shown by decreased Laminb1, PCNA and increased p21, IL-6 expression (Figure 2E-F, Figure S2A-B).

To gain a comprehensive understanding of the pathological effects of chronic HHcy on the corpus cavernosum, we performed single-cell RNA sequencing (Figure 2G-H). Violin plots of senescence-associated gene expression (cell cycle arrest, DDR, SASP) in endothelial cell subpopulations indicated that the transcriptomic profile of ECs in the Mid-old+HHcy group exhibited pronounced senescent alterations (Figure 2J). Monocle2 pseudotime analysis further confirmed that, relative to natural aging, the additional effect of HHcy caused endothelial cells to diverge in different directions and accelerated their transition from pre-aging to senescence, as evidenced by the bifurcation at Branch Point 1 (Figure 2K). Pseudotemporal analysis of Cdkn1a, H2ax, and Il6 revealed that, compared to naturally aging rats, HHcy accelerated the upregulation of these senescence-associated genes (Figure 2I). Collectively, these results provide strong evidence that elevated Hcy levels contribute to endothelial senescence and vascular remodeling, leading to the development of ED in middle-aged rats.

### Hcy accelerates endothelial senescence via MARS1-HTL-protein K-Hcy pathway

**Figure 3:**
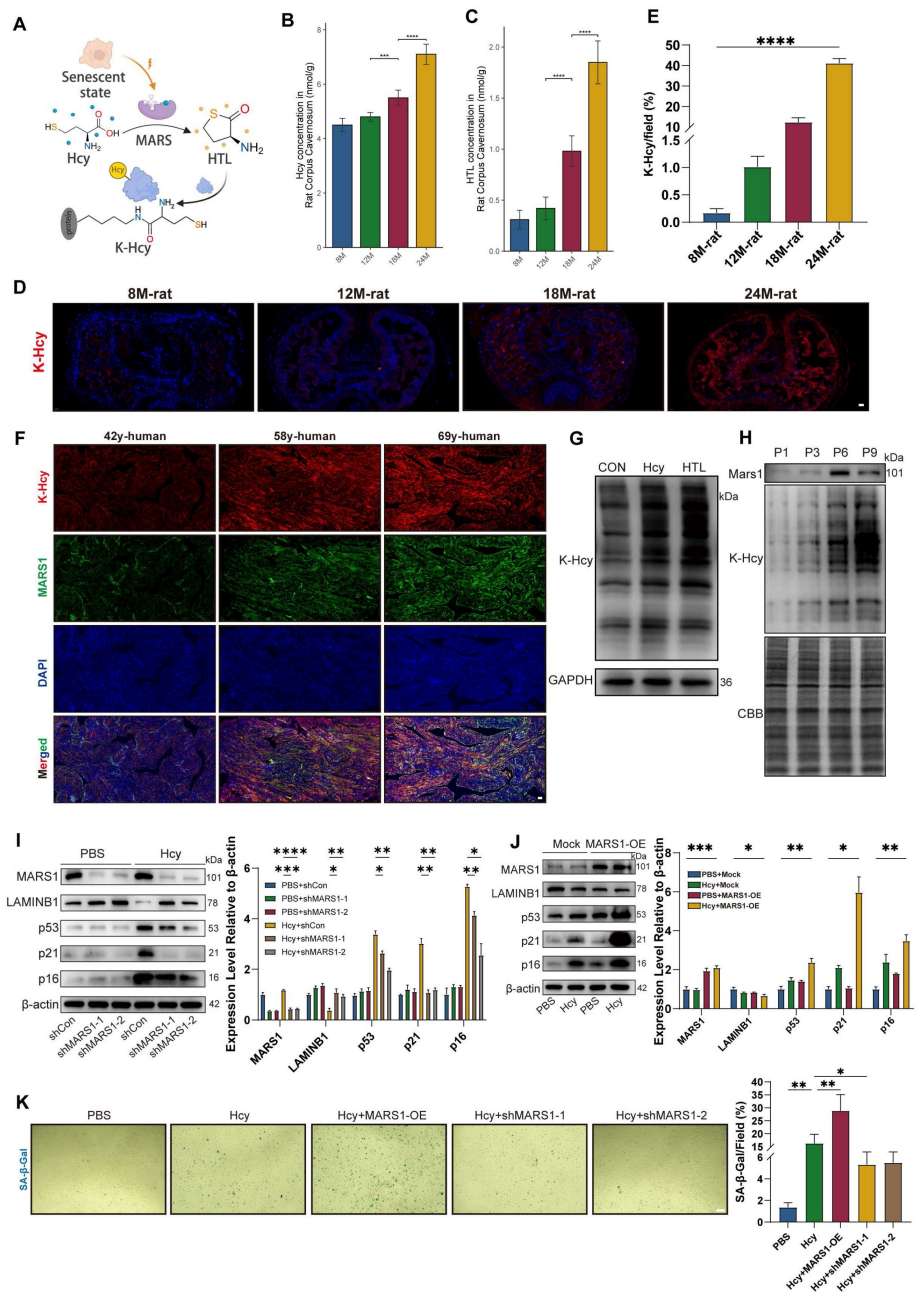
** Hcy accelerates endothelial senescence via MARS1-HTL-protein K-Hcy pathway. A.** Schematic representation of Hcy-induced protein lysine-homocysteinylation (K-Hcy). **B-C.** Age-dependent accumulation of Hcy (B) and HTL (C) in corpus cavernosum of naturally aged rats (n=4/group).** D-E.** Representative immunofluorescence images (D) and semi-quantification (E) of K-Hcy in naturally aged rat penile sections. Scale bars: 100 μm. **F.** Representative immunofluorescence images of K-Hcy and MARS1 co-localization in human corpus cavernosum from physiological Hcy donors. Scale bars: 50 μm.** G.** Representative immunoblot of K-Hcy in passage 2 (P2) rat primary corpus cavernosum endothelial cells (CCECs) treated with Hcy (200 μM) or HTL (100 μM) for 12 h. **H.** Representative immunoblot of MARS1 and K-Hcy in CCECs across population doublings (P1, P3, P6, P9).** I-J.** Western blot analysis of MARS1 and senescence markers (LAMINB1, p53, p21, p16) in HCMECs with MARS1 knockdown (P10-15) (I) or MARS1 overexpression (P3-P8) (J) treated with 200 μM Hcy for 96h. Band intensities were quantified relative to the untreated group (n=3). **K.** Representative images and semi-quantification of SA-β-gal activity in MARS1-knockdown or -overexpression HCMECs treated with 200 μM Hcy for 96h (n=3). Scale bars: 50 μm.

We further explored the potential molecular mechanisms underlying the enhanced pathological effects of Hcy on ED in aging populations. Previous studies in neurodegenerative diseases such as Alzheimer's and Parkinson's have demonstrated that both Hcy and its active derivative, homocysteine thiolactone (HTL), accumulate with age in the brain^16^, ^18^. HTL promotes non-enzymatic N-homocysteinylation (K-Hcy) of proteins, a modification increasingly implicated in the pathogenesis of cardiovascular diseases. Therefore, we hypothesized that Hcy accelerates endothelial senescence via MARS1-HTL-mediated protein K-Hcy pathway (Figure 3A). In naturally aged rats, we observed an age-dependent accumulation of both Hcy and HTL in the corpus cavernosum (Figures 3B-C). Furthermore, immunofluorescence analyses revealed that K-Hcy modifications increased progressively with age in naturally aging rats (Figures 3D-E). In human corpus cavernosum from physiological Hcy donors, robust colocalization of K-Hcy with MARS1 was also observed (Figure 3F). *In vitro*, primary corpus cavernosum endothelial cells (CCECs) treated with either 200 μM Hcy or 100 μM HTL for 12 hours exhibited significantly increased K-Hcy levels (Figure 3G), and a progressive rise in K-Hcy was noted across cell passages, whereas Mars1 expression increased initially and then decreased over successive passages. The subsequent decline in MARS1 expression may reflect metabolic stagnation and reduced protein synthesis demand in overly senescent CCECs (Figure 3H).

To further determine whether MARS1 mediates Hcy-induced endothelial senescence, we manipulated MARS1 expression. Due to difficulties with genetic manipulation in primary CCECs, we employed human cardiac microvascular endothelial cells (HCMECs) at passages 10-15 for MARS1 knockdown experiments, and passages 3-8 for MARS1 overexpression studies. Western blot analysis in HCMECs showed that MARS1 knockdown significantly attenuated the expression of key senescence markers (LAMINB1, p53, p21, and p16) following 96 hours of 200 μM Hcy treatment, whereas MARS1 overexpression enhanced their expression (Figures 3I-3J). This molecular evidence was corroborated by changes in SA-β-gal activity, which was significantly modulated by MARS1 expression levels in HCMECs treated with Hcy (Figure 3K). Collectively, these findings demonstrate that Hcy promotes endothelial senescence through the MARS1-HTL-K-Hcy pathway, with elevated MARS1 and HTL levels in mid-old conditions further amplifying the overall signaling cascade.

### Hcy induces K-Hcy of HMGB1/2 in endothelial cells

**Figure 4:**
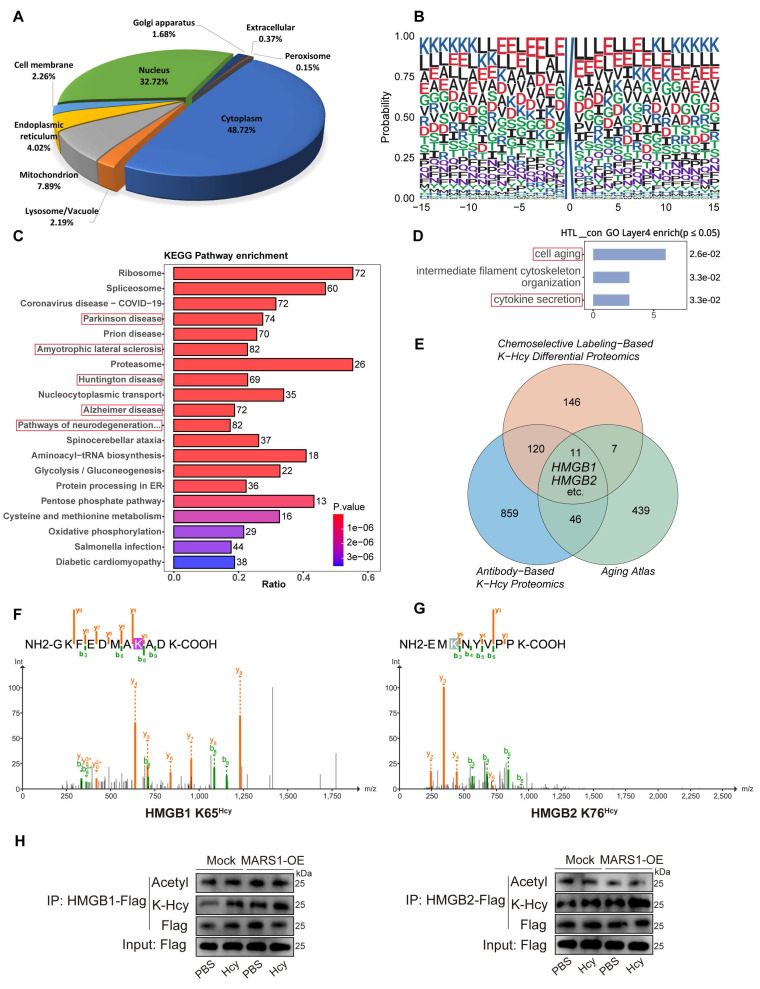
** Hcy induces K-Hcy of HMGB1/2 in endothelial cells. A.** Subcellular distribution of K-Hcy-modified proteins in endothelial cells via anti-K-Hcy proteomics (DeepLoc 2.1-predicted). **B.** Sequence conservation analysis of K-Hcy-targeted motifs. **C.** KEGG pathway enrichment of K-Hcy-modified proteins highlights degenerative disease-associated pathways. **D.** GO biological process enrichment of chemoselective labeling-based K-Hcy differential proteomics. **E.** Venn diagram of K-Hcy proteomic overlap among chemoselective labeling, antibody-based IP, and Aging Atlas. **F-G.** Representative MS/MS spectra validating lysine-homocysteinylation (K-Hcy) at HMGB1 K65 (F) and HMGB2 K76 (G). **H.** Western blot showed that Hcy (200 μM, 96h) treatment and/or MARS1 overexpression enhanced HMGB1/2 K-Hcy in HCMECs, with no effect on acetylation.

To provide further mechanistic insight into how K-Hcy contributes to endothelial senescence, we performed an unbiased antibody-based K-Hcy proteomic analysis, revealing its subcellular distribution in endothelial cells (Figure 4A). Together, we identified 3,816 K-Hcy-modified peptides representing 1,036 distinct proteins (Dataset S1). Sequence conservation analysis revealed distinct K-Hcy-targeted motifs (Figure 4B). KEGG pathway enrichment of these K-Hcy-modified proteins demonstrated associations with multiple degenerative diseases (Figure 4C). We further employed an alternative approach, chemoselective labeling of K-Hcy using biotin-azide, to compare differential protein modifications in endothelial cells with or without Hcy (200 μM) incubation. Western blot analysis confirmed the pH-dependent specificity of this method, consistent with previous reports^35^ (Figure S4A). Through this approach, we identified 284 differentially modified proteins (Dataset S2 and Figure S4D). GO biological process analysis revealed that these proteins were enriched in key pathways such as *cell aging* and *cytokine secretion* (Figure 4D). Overlapping analyses among antibody-based, chemoselective labeling-based K-Hcy proteomics, and the Aging Atlas^40^ confirmed a subset of conserved K-Hcy-modified proteins, including High mobility group box-1 and 2 (HMGB1/2) (Figure 4E).

HMGB1/2 are essential and highly conserved regulators of genomic stability, with their functions widely reported to be modulated by lysine acetylation^41^, suggesting that K-Hcy may similarly impact their biological functions (Figure S4E). Therefore, we further incubated HCMECs overexpressing Flag-HMGB1/2 with Hcy and performed immunoprecipitation (IP)-LC-MS/MS to screen for K-Hcy-modified sites in HMGB1/2. The results revealed that multiple lysine residues in HMGB1/2 underwent varying degrees of K-Hcy modification (Figure S4F and Dataset S3), with HMGB1 K12, K65, and K68, as well as HMGB2 K12, K65, K76, and K157, exhibiting higher modification levels (modification/base ratio >0.5) (Figure 4F-G and Figure S4B-C). Moreover, western blot analysis showed that Hcy treatment (200 μM, 96h) and/or MARS1 overexpression significantly enhanced HMGB1/2 K-Hcy levels in HCMECs, with no observed changes in acetylation (Figure 4H). These results establish HMGB1/2 as critical K-Hcy targets in endothelial cells, linking K-Hcy modification to endothelial senescence.

### K-Hcy drives nuclear-to-cytoplasmic translocation of HMGB1/2

**Figure 5:**
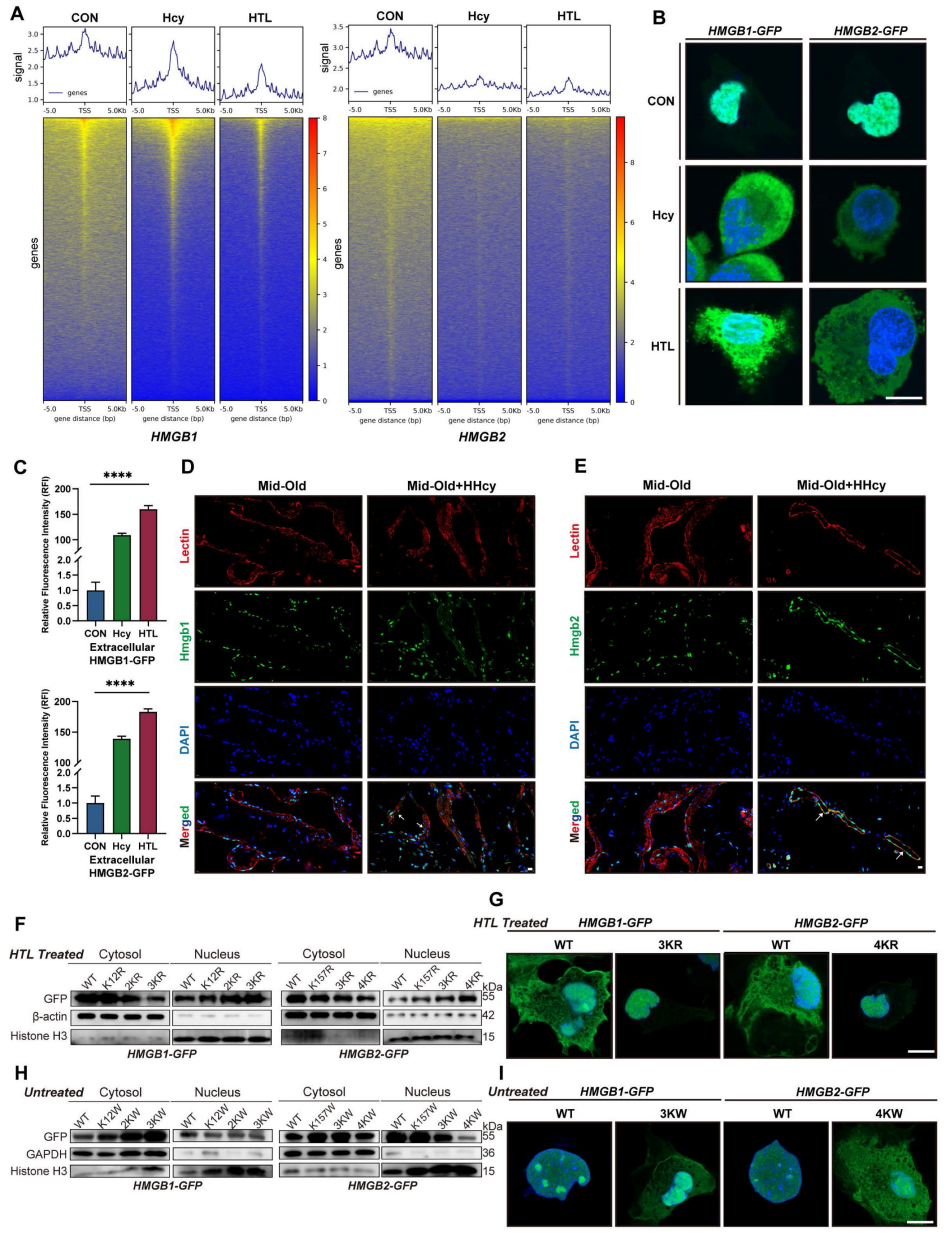
** K-Hcy drives nuclear-to-cytoplasmic translocation of HMGB1/2. A.** Cut&Tag TSS heatmaps of HMGB1/2 in HCMECs treated with Hcy (200 μM) or HTL (100 μM) for 96h, showing altered DNA-binding signals. **B.** Confocal fluorescence images demonstrated Hcy/HTL-induced nuclear-to-cytoplasmic translocation of HMGB1/2-GFP in HCMECs (Hcy: 200 μM; HTL: 100 μM; 96h). Scale bars: 10 μm. **C.** Quantification of extracellular HMGB1/2-GFP fluorescence intensity (n=3). **D-E.** HMGB1/2 (green) and Lectin (red) co-localization in rat penile sections (Mid-old/Mid-old+HHcy), with DAPI-counterstained nuclei (blue). White arrows indicate nuclear-to-cytoplasmic translocation of HMGB1/2. Scale bars: 10 μm. **F.** Cytosolic/nuclear fractionation immunoblots of HMGB1/2-GFP wild-type (WT) and lysine-to-arginine mutants in HTL-treated HCMECs. HMGB1 mutations: 2KR (K65,68R), 3KR (K12,65,68R). HMGB2 mutations: 3KR (K12,65,76R), 4KR (K12,65,76,157R). Normalized to β-actin (cytosol) and Histone H3 (nucleus). **G.** Representative confocal images of corresponding GFP-tagged constructs, relative to F. **H.** Cytosolic/nuclear fractionation immunoblots of HMGB1/2-GFP WT and lysine-to-tryptophan mutants in HCMECs. HMGB1 mutations: 2KW (K65,68W), 3KW (K12,65,68W). HMGB2 mutations: 3KW (K12,65,76W), 4KW (K12,65,76,157W). Normalized to GAPDH (cytosol) and Histone H3 (nucleus).** I.** Representative confocal images of corresponding GFP-tagged constructs, relative to H.

We further investigate how K-Hcy impacts the biological function of HMGB1/2. Under physiological conditions, HMGB1/2 are primarily localized in the nucleus, where they bind to DNA and contribute to nucleosome structural integrity. Therefore, we first evaluated changes in HMGB1/2 DNA binding upon Hcy or HTL treatment using Cut&Tag assays. The results revealed a significant reduction in overall HMGB1/2 DNA-binding signals in HCMECs (Figure 5A). We speculated that this phenomenon might result from nuclear depletion of HMGB1/2 through a translocation mechanism, a well-recognized hallmark of cellular senescence. To test this hypothesis, we transfected HCMECs with HMGB1/2-GFP fusion constructs and examined their subcellular localization. Confocal microscopy revealed a pronounced nuclear-to-cytoplasmic translocation of HMGB1/2-GFP following Hcy or HTL exposure (Figure 5B), with extracellular fluorescence intensity quantification further confirming increased HMGB1/2 secretion (Figure 5C). In vivo, immunofluorescence staining of rat penile sections also showed enhanced cytoplasmic HMGB1/2 localization in mid-old+HHcy conditions (Figure 5D-E).

The next critical question to address is whether K-Hcy directly mediates this translocation. To investigate this, we generated lysine-to-arginine (K→R) and lysine-to-tryptophan (K→W) HMGB1/2 mutants, which prevent or mimic K-Hcy, respectively. Cytosolic/nuclear fractionation western blotting first demonstrated that HMGB1 K12,65,68R (3KR) and HMGB2 K12,65,76,157R (4KR) mutations did not alter their nuclear localization under basal conditions (Figure S5C). However, upon HTL treatment, WT HMGB1/2 exhibited substantial cytoplasmic translocation, whereas K→R mutants remained predominantly nuclear (Figure 5F). Confocal imaging further corroborated these observations (Figure 5G). In contrast, K→W mutants were designed to mimic the bulky side chain effects of K-Hcy. AlphaFold3-predicted 3D structural modeling suggested that K→W substitutions alter both the binding affinity and interaction pattern between HMGB1/2 and Importin Alpha 5 (IMA5, a key nuclear import mediator for HMGB1/2) (Figure S5A-B). Western blot and confocal imaging further confirmed the cytoplasmic retention of K→W mutants (Figure 5H-I). These findings collectively indicated that K-Hcy directly drives the nuclear-to-cytoplasmic translocation of HMGB1/2, thereby mediating nuclear depletion of HMGB1/2 under Hcy exposure.

### K-Hcy-modified extracellular HMGB1/2 enhances SASP by attenuating SLIT2-ROBO4 anti-inflammatory signaling

**Figure 6:**
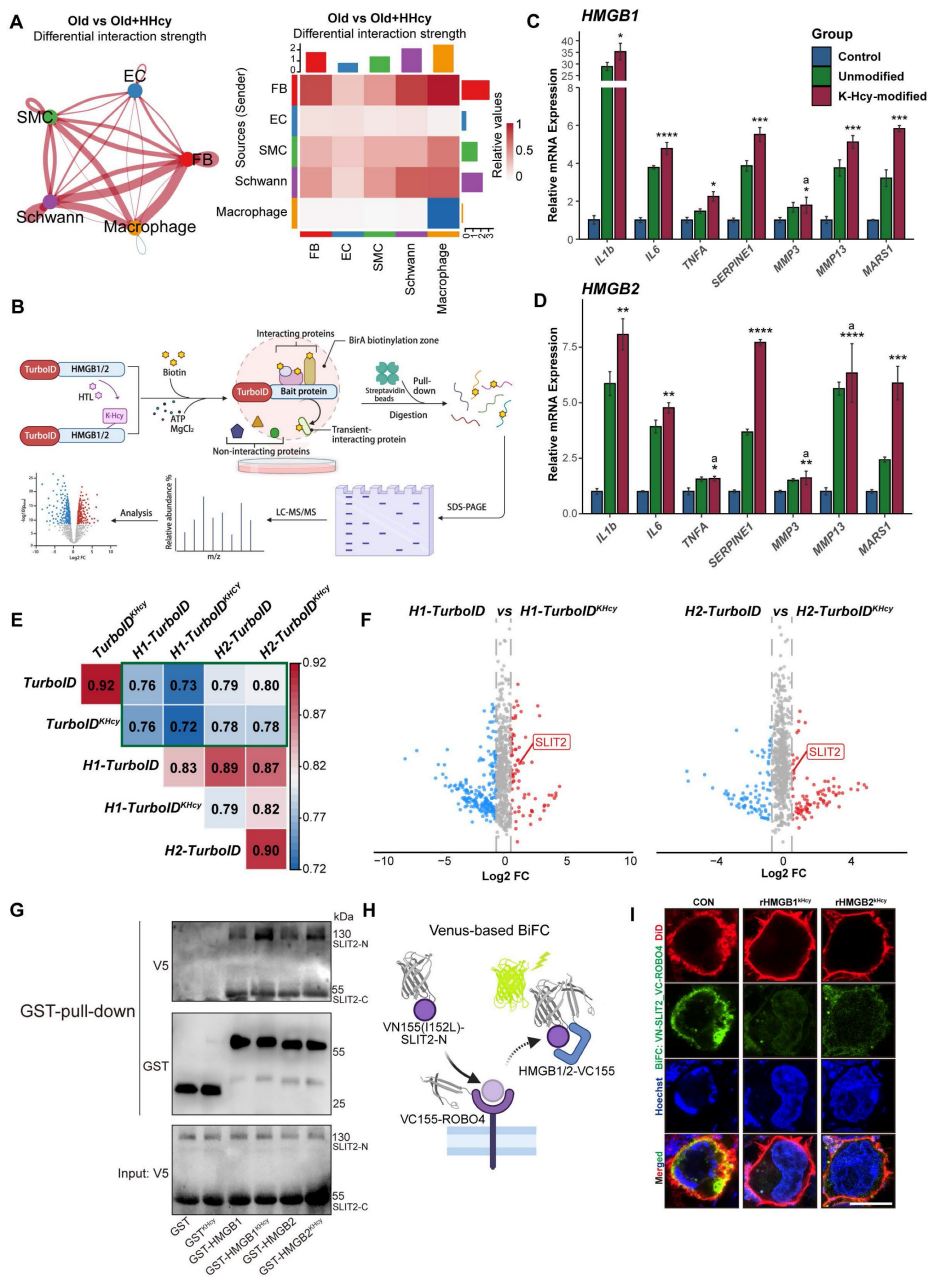
** K-Hcy-modified extracellular HMGB1/2 enhances SASP by attenuating SLIT2-ROBO4 anti-inflammatory signaling. A.** CellChat analysis of single-cell transcriptomics reveals significantly enhanced global cell-cell communication in Mid-old+HHcy group compared to Mid-old controls. **B.** TurboID-based proximity labeling workflow for mapping K-Hcy-modified HMGB1/2 interactomes: biotinylation (1h), streptavidin pulldown, and LC-MS/MS identification. **C-D.** qRT-PCR analysis of SASP markers and *MARS1* in HCMECs treated for 24h with K-Hcy-modified or unmodified recombinant HMGB1/2 (1 μg/mL) (*n* = 3). **E.** Correlation heatmap of TurboID interactomes comparing HMGB1/2-TurboID groups (±K-Hcy modification) with background controls (TurboID-only). Hierarchical clustering shows weak correlation between TurboID-only and HMGB1/2-TurboID groups. **F.** Volcano plot of differential interactome protein intensity between HMGB1/2-TurboID and HMGB1/2-TurboID^KHcy^ groups (background-subtracted), highlighting SLIT2 as the potential candidate. **G.** GST pull-down assay confirms direct binding of HMGB1/2 to SLIT2-N terminal (N) and SLIT2-C terminal (C), with K-Hcy modification enhancing HMGB1/2 and SLIT2-N interaction strength. **H.** Schematic model illustrating HMGB1/2-dependent modulation of SLIT2-ROBO4 interaction, as demonstrated by the Venus-based BiFC assay. **I.** BiFC assay in 293T cells co-expressing VN155 (I152L)-SLIT2 and VC155-ROBO4 plasmids, with/without recombinant K-Hcy-modified HMGB1/2 stimulation (1 μg/mL, 24h). DiD (red): cell membrane staining; BiFC signal (green); Hoechst (blue): nuclei. Scale bars: 10 μm.

K-Hcy modification enhanced the extracellular release of HMGB1/2 and this paracrine senescence signal may amplify SASP and its impact on surrounding cells. Therefore, we further explored the biological function of K-Hcy-modified extracellular HMGB1/2. Leveraging previously acquired single-cell transcriptomic data from the corpus cavernosum, CellChat analysis revealed a significant increase in global cell-cell communication in the Mid-old+HHcy group compared to Mid-old controls (Figure 6A). qRT-PCR analysis revealed that treatment with K-Hcy-modified recombinant HMGB1/2 significantly upregulated both SASP markers and MARS1 expression in HCMECs, suggesting a potential HMGB1/2^K-Hcy^-MARS1-driven positive feedback loop and an intensified paracrine senescence signaling cascade (Figure 6C-D). To explore the molecular mechanisms underlying the enhancement of SASP by K-Hcy-modified extracellular HMGB1/2, we utilized TurboID-based proximity labeling, followed by streptavidin pulldown and LC-MS/MS analysis (Figure 6B). WB and flow cytometry analysis confirmed the effectiveness of the recombinant HMGB1/2-TurboID-based proximity labeling system (Figure S6A-B). Correlation analysis of TurboID interactomes demonstrated a clear separation between HMGB1/2-TurboID groups (±K-Hcy) and background controls (Figure 6E). Interestingly, differential interactome analysis identified multiple secreted proteins, including Slit Guidance Ligand 2 (SLIT2), an important anti-inflammatory factor secreted by endothelial cells (Figure 6F and Dataset S4). These findings suggested that K-Hcy-modified HMGB1/2 may destabilize the equilibrium between pro-inflammatory and anti-inflammatory cytokine networks by interacting with SLIT2.

To validate this interaction, GST pull-down assays were performed and confirmed direct binding of HMGB1/2 to SLIT2-N terminal (N) and SLIT2-C terminal (C), with K-Hcy modification enhancing HMGB1/2 and SLIT2-N interaction strength. (Figure 6G). Consistently, Venus-based bimolecular fluorescence complementation (BiFC) assays in 293T cells overexpressing VN155 (I152L)-SLIT2-N and HMGB1/2-VC155 showed that HTL treatment induced significant co-localization of BiFC signals with the cell membrane, indicating that extracellular HMGB1/2 interacts with SLIT2 following its release (Figure S6C-D). Given that SLIT2-ROBO4 signaling is a critical anti-inflammatory pathway in endothelial cells^42^, ^43^, we next examined whether K-Hcy-modified HMGB1/2 influences this axis. BiFC assays revealed that K-Hcy-modified HMGB1/2 disrupted SLIT2-ROBO4 interactions, as evidenced by reduced BiFC signals upon recombinant K-Hcy-HMGB1/2 treatment in 293T cells co-expressing VN155(I152L)-SLIT2-N and VC155-ROBO4 (Figure 6H-I). Additionally, qRT-PCR analysis revealed that K-Hcy-modified HMGB1/2 significantly suppressed SLIT2 mRNA expression in endothelial cells (Figure S6E). Together, these findings indicated that K-Hcy-modified extracellular HMGB1/2 promotes SASP by interfering with SLIT2-ROBO4-mediated anti-inflammatory signaling, thereby exacerbating endothelial paracrine senescence.

### N-acetylcysteine attenuates endothelial senescence and rescues erectile function in middle-aged HHcy rat by blocking K-Hcy

**Figure 7:**
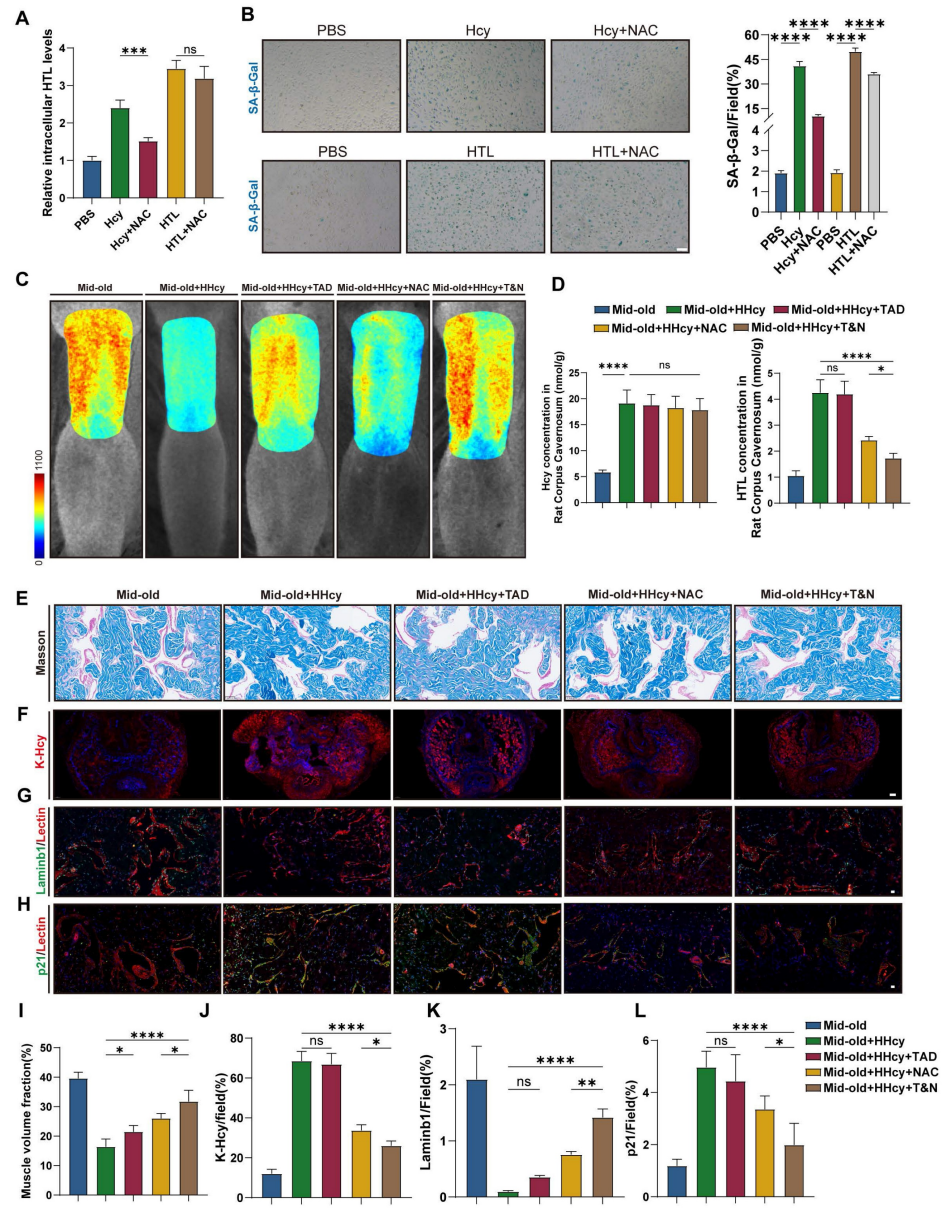
** N-acetylcysteine attenuates endothelial senescence and rescues erectile function in middle-aged HHcy rat by blocking K-Hcy. A.** N-Acetylcysteine (NAC) decreased intracellular HTL levels induced by homocysteine. CCECs were treated with 200 μM Hcy, 100 μM HTL and 1 mM NAC for 12 h before harvesting (n=4). **B.** Representative images and semi-quantification of SA-β-gal activity in CCECs treated with Hcy (200 μM) and NAC (2 mM) for 96h (n=4). Scale bars: 50 μm. **C.** Representative laser speckle contrast imaging of sodium nitroprusside (NO donor)-induced rat penile blood flow across five experimental groups (n=3): Mid-old, Mid-old+HHcy, Mid-old+HHcy+Tadalafil (TAD), Mid-old+HHcy+NAC and Mid-old+HHcy+Tadalafil & NAC (T&N). **D.** Hcy and HTL levels in rat corpus cavernosum tissues across all experimental groups (*n* = 4). **E, I.** Representative images and semi-quantification of Masson's trichrome-stained rat corpus cavernosum sections (n=4). Scale bars: 50 μm. **F, J.** Representative immunofluorescence images and semi-quantification of K-Hcy in rat penile sections. Scale bars: 100 μm. **G, K.** Representative immunofluorescence co-staining images and semi-quantification in different groups (n=4). Laminb1 (green) and Lectin (red). **H, L.** Representative immunofluorescence co-staining images and semi-quantification in different groups (n=4). p21 (green) and Lectin (red). Scale bars: 20 μm.

Finally, we tested whether pharmacological blocking K-Hcy could ameliorate endothelial senescence in the corpus cavernosum and rescue erectile function in middle-aged HHcy rats. N-Acetylcysteine (NAC), a well-known antioxidant, can competitively inhibit MARS1^44^. While NAC treatment markedly attenuated the elevation of intracellular HTL levels induced by Hcy in CCECs, it had no impact on intracellular HTL levels under direct HTL exposure. (Figure 7A). Moreover, NAC effectively attenuated Hcy-induced senescence, as indicated by reduced SA-β-gal activity in CCECs. Interestingly, an anti-senescent effect was also observed under HTL stimulation, potentially due to NAC's intrinsic antioxidant capacity beyond its impact on Hcy metabolism (Figure 7B). To assess erectile function, we performed laser speckle contrast imaging and ICP measurements. The results showed that NAC-treated rats exhibited increased blood flow compared to the Mid-old+HHcy group. Although the improvement was less pronounced than that observed with tadalafil alone, the combined NAC and tadalafil (T&N) treatment led to a further enhancement (Figure 7C and Figure S7A). Consistently, corpus cavernosum HTL levels were significantly reduced in the NAC or T&N-treated groups, whereas no notable change was observed in the tadalafil-treated group (Figure 7D).

Histological analysis further confirmed the protective effects of NAC, while the combined treatment with NAC and tadalafil provided additional benefits. Masson's trichrome staining showed a reduction in collagen-to-smooth muscle ratio in the corpus cavernosum of NAC-treated rats, with a further decrease observed in the T&N group (Figure 7E, I). Immunofluorescence analysis revealed that NAC treatment markedly decreased K-Hcy accumulation in penile tissues (Figure 7F, J). Furthermore, endothelial senescence was alleviated in NAC-treated groups, as evidenced by an increase in LaminB1-positive endothelial cells (Figure 7G, K) and a reduction in p21 expression, with the most pronounced improvement observed in the T&N group (Figure 7H, L). Collectively, these results indicated that in the context of middle-aged HHcy, NAC effectively alleviates endothelial senescence in the corpus cavernosum, preserves cavernous structural integrity by blocking K-Hcy, enhances erectile function, and potentiates the therapeutic efficacy of tadalafil.

## Discussion

In this study, we integrated clinical observations, animal experiments, and cellular assays to establish that HHcy accelerates cavernous endothelial senescence, precipitating ED in middle-aged and elderly populations. Clinically, we demonstrated that plasma Hcy levels are modestly associated with ED risk in younger men but surge in their pathological impact after middle age, a phenomenon mirrored in our rat models. This age-dependent amplification of Hcy toxicity is driven by the progressive upregulation of the MARS1-HTL-K-Hcy axis during natural aging: elevated MARS1 in mid-old endothelial cells boosts HTL synthesis and K‑Hcy modification of proteins HMGB1/2. K-Hcy modification drives nuclear-to-cytoplasmic translocation and extracellular release of HMGB1/2, thereby amplifying paracrine senescence signaling through enhanced SASP. Importantly, pharmacological blockade of MARS1 with N‑acetylcysteine effectively attenuated endothelial senescence and rescued erectile function, highlighting the therapeutic promise of targeting homocysteine sensing for treating age-exacerbated ED in HHcy populations.

HHcy is highly prevalent in older adults, affecting up to half of men over 60^45^, and parallels a steep rise in ED incidence after middle age. Although ED has traditionally been viewed as a sexual health issue, it is now recognized as an early sentinel for cardiovascular disease (CVD). Hcy is an established independent risk factor in atherosclerosis, hypertension, thrombotic disorders, and various other CVDs^46^, yet its direct mechanistic role in ED has remained elusive. Folate supplementation reliably lowers plasma Hcy; in combination with tadalafil, it has been shown in meta-analyses to improve IIEF-5 scores. However, the degree to which this benefit derives from Hcy reduction remains uncertain^47^. Moreover, clinical trials of folate or vitamins B6/B12 in cardiovascular cohorts with HHcy have produced inconsistent or negligible therapeutic effects^48^. These paradoxes suggest that the pathological impact of Hcy in ED is driven by other downstream mediators—such as HTL‑induced K‑Hcy modifications—that are not ameliorated by Hcy‑lowering strategies alone.

Emerging research has shifted focus to HTL, a reactive metabolite exclusively generated from Hcy by MARS1 misacylation. MARS1 is essential for protein translation, highly expressed in developing and proliferating cells, and also upregulated by high‑fat diets through palmitate‑induced NF‑κB activation^28^, ^49^. Moreover, interindividual variations in baseline MARS1 expression may underlie the remarkable heterogeneity in plasma and urinary HTL levels, which can fluctuate by more than a thousand-fold across populations^48^. In this study, we found that naturally aged corpus cavernosum exhibit significantly elevated HTL and K‑Hcy levels compared to young counterparts, alongside increased MARS1 expression in mid‑passage endothelial cells. These observations, mirroring K‑Hcy dynamics reported in Parkinson's disease models^18^, indicate that the aging endothelium has an enhanced capacity for HTL synthesis, thereby amplifying the pathological effects of Hcy in middle‑aged ED.

Protein post-translational modifications (PTMs) have emerged as important regulators of penile hemodynamics and the vascular pathophysiology of ED. Diverse PTMs finely modulate signaling pathways critical for cavernosal smooth muscle relaxation and endothelial function. For instance, cyclic AMP-dependent phosphorylation of nNOS directly promotes penile erection, while eNOS phosphorylation and S-nitrosylation control nitric oxide bioavailability^50^-^52^. Conversely, dysregulation of PTMs impairs cavernosal vascular function, promoting the onset and progression of ED. In the context of diabetes, chronic hyperglycemia disrupts glycosylation homeostasis, and glycosylated serum protein levels have been shown to predict endothelial dysfunction and ED independently of conventional risk factors^53^. Notably, Musicki and colleagues demonstrated that O-GlcNAcylation of eNOS at Ser-1177 prevents its activation by shear stress and VEGF signaling, highlighting a direct mechanistic link between PTM dysregulation and penile vascular dysfunction^54^. Additionally, dysregulation of ubiquitination, a key mechanism for protein turnover, contributes to ED pathophysiology. Our previous study showed that high glucose promotes GPX4 ubiquitination via the E3 ligase NEDD4, leading to increased intracellular oxidative stress and enhanced ferroptosis^55^. Beyond these classical PTMs, K‑Hcy represents a pathological, non‑enzymatic PTM that occurs in direct proportion to cellular Hcy and HTL concentrations and has emerged as a key mediator of protein dysfunction, cytotoxicity, inflammation, and atherothrombosis^15^. It is increasingly recognized in both cardiovascular and neurodegenerative disorders, where K‑Hcy of proteins such as α‑synuclein and DJ‑1 drives aggregation and toxicity^17^, ^18^, ^56^. In our study, two complementary K‑Hcy proteomic strategies generated a comprehensive modification map and revealed that endothelial HMGB1/2 are heavily K‑Hcy‑modified targets. Furthermore, KEGG pathway enrichment of K‑Hcy‑modified proteins highlighted links to multiple neurodegenerative disease pathways. Notably, prior non‑endothelial K‑Hcy proteomic analyses also identified HMGB1/2, underscoring the evolutionary conservation of their modification^44^, ^57^.

HMGB1/2, which are highly evolutionarily conserved and play crucial roles in chromatin remodeling, DNA repair, and transcriptional regulation, undergo nuclear depletion—a hallmark of senescence. HMGB1/2 can be released extracellularly via active or passive mechanisms. Our previous work demonstrated that extracellular HMGB1 exacerbates inflammation and endothelial dysfunction in diabetic corpus cavernosum through the receptor for advanced glycation end products (RAGE)/NF-κB signaling pathway^58^. HMGB1 has also been elegantly implicated in kidney-related disorders, particularly in CKD, where it contributes to inflammation, fibrosis, aging, and vascular calcification^59^. Beyond its well-established proinflammatory actions, HMGB1 functions as a redox-sensitive protein that dimerizes under oxidative stress to protect adjacent DNA from hydroxyl radical-induced damage^60^. Endogenous HMGB1 is also a critical regulator of autophagy, with its intramolecular disulfide bridge (C23/45) being essential for Beclin1 binding and autophagy maintenance^61^. Compared with HMGB1, HMGB2 is less well characterized. Despite their high sequence and structural similarity, the two proteins differ markedly in expression and function. Unlike HMGB1, loss of HMGB2 alone is not sufficient to induce aging^62^; however, emerging evidence indicates that HMGB2 depletion disrupts higher-order chromatin organization, potentially constituting an initiating event in the aging program across diverse cell types^21^. Moreover, HMGB1/2 are regulated by various PTMs, with lysine acetylation being the most extensively studied. Our study clearly demonstrated that under Hcy conditions, HMGB1/2 undergo nuclear-cytoplasmic translocation and extracellular release, independent of acetylation but mediated by K-Hcy modification, thereby linking Hcy metabolism directly to endothelial aging.

Paracrine senescence is predominantly mediated by SASP, through which senescent cells release a spectrum of inflammatory cytokines (e.g., IL-6, IL-1β, TNF-α), growth factors, and proteases. These SASP components activate multiple signaling cascades, including the NF-κB pathway, in neighboring cells, thereby propagating senescence and creating a pro-inflammatory microenvironment conducive to age-related pathologies^63^. Inflammation is a fundamental mechanism contributing to vascular dysfunction, affecting both endothelial and smooth muscle cells, as well as age-related changes that ultimately predispose to ED and CVD^64^. In metabolic disorder-associated ED, such as that linked to diabetes and obesity, elevated circulating inflammatory cytokines, chemokines (e.g., IL-1), and adhesion molecules (ICAM-1, VCAM-1, and P-selectin) play pivotal roles in disease progression. These factors disrupt intercellular communication within the corpus cavernosum and reduce nitric oxide (NO) bioavailability, thereby exacerbating endothelial dysfunction and ultimately impairing erectile function^65^. Our study demonstrates that K-Hcy-modified HMGB1/2 act as extracellular enhancers of endothelial SASP. As a well-documented damage-associated molecular pattern (DAMP), HMGB1 engages Toll-like receptors (TLRs) and RAGE, amplifying inflammatory cascades and perturbing endothelial homeostasis^66^. Utilizing TurboID proximity labeling, GST pull-down, and BiFC assays, we identified a novel interaction between K-Hcy-HMGB1/2 and the anti-inflammatory secreted protein SLIT2. This interaction disrupts SLIT2-ROBO4 signaling, skewing the senescent microenvironment toward pro-inflammatory dominance. Notably, SLIT2—a youth-associated factor downregulated in aging—has previously been shown to reverse mid-old-related functional decline^67^, underscoring the pathophysiological significance of its inhibition by K-Hcy-HMGB1/2. Furthermore, K-Hcy-HMGB1/2 upregulates MARS1 mRNA, suggesting a feedforward loop that perpetuates senescence. These findings elucidate the dynamic interplay of extracellular signaling molecules in mediating paracrine senescence. Due to the non-enzymatic nature of K-Hcy modifications, extracellular proteins are also susceptible. A notable example involves low-density lipoprotein (LDL), which undergoes HTL-mediated modification to form K-Hcy-LDL. This modified LDL exhibits enhanced uptake by vascular endothelial cells and macrophages, driving oxidative stress and accelerating atherosclerosis progression^68^. Oxidative stress, a well-established pathogenic factor in ED, arises from an imbalance between ROS production and antioxidant defenses. Mechanistically, the pro-oxidant nature of Hcy is largely attributed to the metal-catalyzed oxidation of thiol-containing molecules, leading to disulfide formation and ROS generation^69^. In contrast, Hcy has also been shown to downregulate glutathione peroxidase 7 (GPx7)-mediated antioxidant pathways, thereby further weakening cellular redox defenses^70^. Moreover, emerging evidence suggests that K-Hcy-modified proteins exhibit heightened vulnerability to oxidative damage^15^. While our study focused on endothelial K-Hcy-HMGB1/2-mediated senescence, K-Hcy modifications may constitute a novel pathological axis that disrupts multiple proteins and cellular processes, thereby driving ED through broader and previously unrecognized mechanisms. Future research should map K-Hcy-modified secretomes and other intracellular proteins in corpus cavernosum to provide a more comprehensive understanding of how K-Hcy contributes to ED.

Collectively, our findings revealed that age-dependent upregulation of MARS1 enhances its sensitivity to Hcy signals, driving site-specific K-Hcy modification of HMGB1/2. K-Hcy promotes HMGB1/2 nuclear-to-cytoplasmic translocation, extracellular release, and subsequent SASP amplification, thereby accelerating endothelial senescence in the corpus cavernosum and contributing to erectile dysfunction in middle-aged and elderly individuals. Pharmacological inhibition of MARS1 with NAC mitigated ED in the middle-aged HHcy populations and potentiated tadalafil's efficacy, underscoring the therapeutic potential of targeting this pathway in HHcy-related ED.

### Limitations of the study

A key limitation of this study is the small sample size in the > 60 years age group (n = 38), which may limit the precision of conclusions for this population. Future studies will aim to increase the sample size in this group and conduct multicenter prospective cohort studies to confirm and strengthen our conclusions. Additionally, we did not include hypertension status or the use of antihypertensive medications as covariates due to incomplete data on blood pressure measurements and medication details. Future studies will address this limitation by capturing more comprehensive data to enhance the robustness of the findings.

## Supplementary Material

Supplementary figures and tables, data.

## Figures and Tables

**Table 1:** Overall baseline characteristics of retrospective age-stratified matched Case-Control study: ED patients (N = 240) and controls (N = 240). Continuous variables are presented as median [interquartile range]. Hcy levels were significantly elevated in ED patients compared to controls (14.4 [11.5;23.0] vs. 12.7 [10.0;15.8] μmol/L, *p* < 0.001).

Overall Summary Descriptive Table			
	CON	ED	p.overall
	*N=240*	*N=240*	
Hcy	12.7 [10.0;15.8]	14.4 [11.5;23.0]	<0.001
BMI	23.8 [21.7;26.3]	23.9 [21.5;25.9]	0.958
TG	1.34 [0.95;1.94]	1.23 [0.86;1.86]	0.152
TC	4.17 [3.61;4.68]	4.18 [3.61;4.65]	0.818
LDL_C	2.67 [2.20;3.10]	2.60 [2.01;3.11]	0.393
HDL_C	1.06 [0.90;1.24]	1.06 [0.90;1.22]	0.928
HbA1c	5.55 [5.38;5.75]	5.50 [5.30;5.71]	0.278
Glu	5.22 [4.87;5.68]	5.21 [4.84;5.74]	0.762
age	39.5 [34.0;53.0]	39.0 [34.0;54.0]	0.801
smoking_history:			0.635
No	216 (90.0%)	220 (91.7%)	
Yes	24 (10.0%)	20 (8.33%)	

**Table 2:** Age-stratified summary descriptive table of case-control study: ≤40 years (N=131 each), 41-60 years (N=71 each), and >60 years (N=38 each). Data presented as median [interquartile range] for non-normally distributed variables and mean (±SD) for normally distributed variables. Categorical variables (smoking history) expressed as frequency.

Age-Stratified Summary Descriptive Table									
	≤40			41-60			>60		
	CON	ED	p.overall	CON	ED	p.overall	CON	ED	p.overall
	*N=131*	*N=131*		*N=71*	*N=71*		*N=38*	*N=38*	
Hcy	12.4 [9.85;15.8]	13.8 [11.1;20.9]	0.016	12.7 [10.3;15.3]	13.8 [11.3;21.2]	0.016	13.3 [11.1;16.4]	23.4 [20.9;29.6]	<0.001
BMI	23.3 [21.2;25.9]	23.5 [20.9;26.0]	0.856	24.4 [23.3;26.5]	24.0 [22.2;25.7]	0.127	23.1 (3.23)	24.5 (2.95)	0.054
TG	1.20 [0.87;1.81]	1.10 [0.76;1.50]	0.088	1.62 [1.18;2.24]	1.38 [1.06;2.12]	0.089	1.19 [0.96;1.53]	1.52 [1.06;2.14]	0.050
TC	4.24 [3.68;4.75]	4.15 [3.54;4.63]	0.294	4.28 (0.68)	4.36 (0.93)	0.555	3.83 [3.25;4.39]	3.96 [3.65;4.50]	0.271
LDL_C	2.73 [2.29;3.22]	2.59 [2.01;3.10]	0.115	2.67 (0.62)	2.62 (1.00)	0.772	2.35 (0.76)	2.58 (0.83)	0.204
HDL_C	1.11 [0.97;1.28]	1.05 [0.94;1.23]	0.121	0.98 [0.86;1.13]	1.08 [0.90;1.25]	0.015	0.99 [0.88;1.15]	0.99 [0.83;1.14]	0.697
HbA1c	5.50 [5.30;5.70]	5.40 [5.30;5.65]	0.102	5.60 [5.40;5.72]	5.50 [5.30;5.70]	0.472	5.71 (0.45)	5.82 (0.42)	0.273
Glu	5.14 [4.81;5.55]	5.07 [4.72;5.60]	0.391	5.33 (0.54)	5.44 (0.59)	0.244	5.56 [5.08;6.30]	5.29 [5.02;6.03]	0.386
age	34.0 [31.0;37.0]	35.0 [32.0;37.0]	0.491	50.0 [45.0;55.0]	50.0 [44.0;54.0]	0.693	67.0 [63.0;71.5]	68.5 [63.0;73.8]	0.542
smoking_history:			0.569			0.743			0.608
No	123 (93.9%)	126 (96.2%)		67 (94.4%)	65 (91.5%)		26 (68.4%)	29 (76.3%)	
Yes	8 (6.11%)	5 (3.82%)		4 (5.63%)	6 (8.45%)		12 (31.6%)	9 (23.7%)	
