# Supplementary material for: N-Homocysteinylation of HMGB1/2 Promotes Corpus Cavernosum Endothelial Senescence in Erectile Dysfunction
Source: Int J Biol Sci. 2025 Oct 20;21(15):6723–44. doi: 10.7150/ijbs.119514 (PMC12631104; doi:10.7150/ijbs.119514)
Supplement: Supplementary file 1 — Supplementary figures and tables, data. [file ijbsv21p6723s1.zip › Supplementary materials/Supplementary materials.pdf]

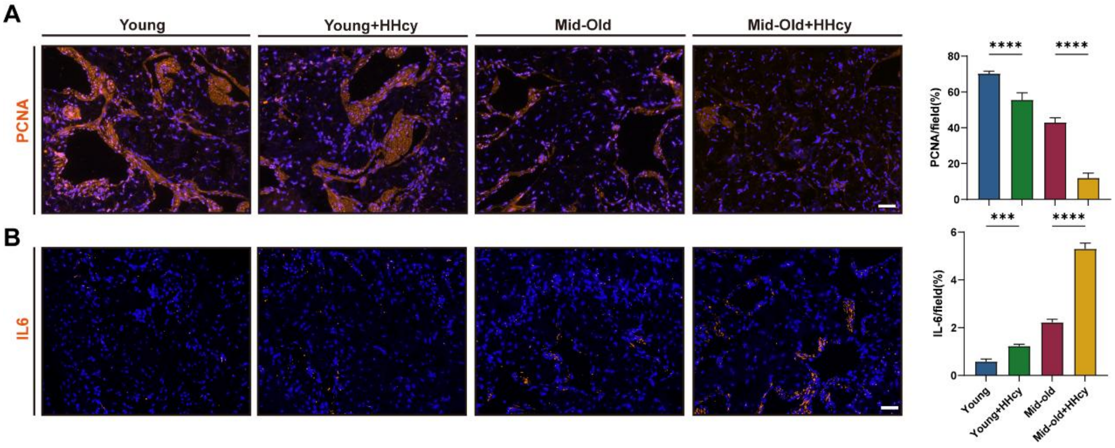

Figure S2. HHcy induces ED in middle-aged rats via accelerated endothelial senescence, related to Figure 2.

A-B. Representative immunofluorescence co-staining images and semi-quantification in different groups: **A.** PCNA (orange). **B.** IL6 (orange).

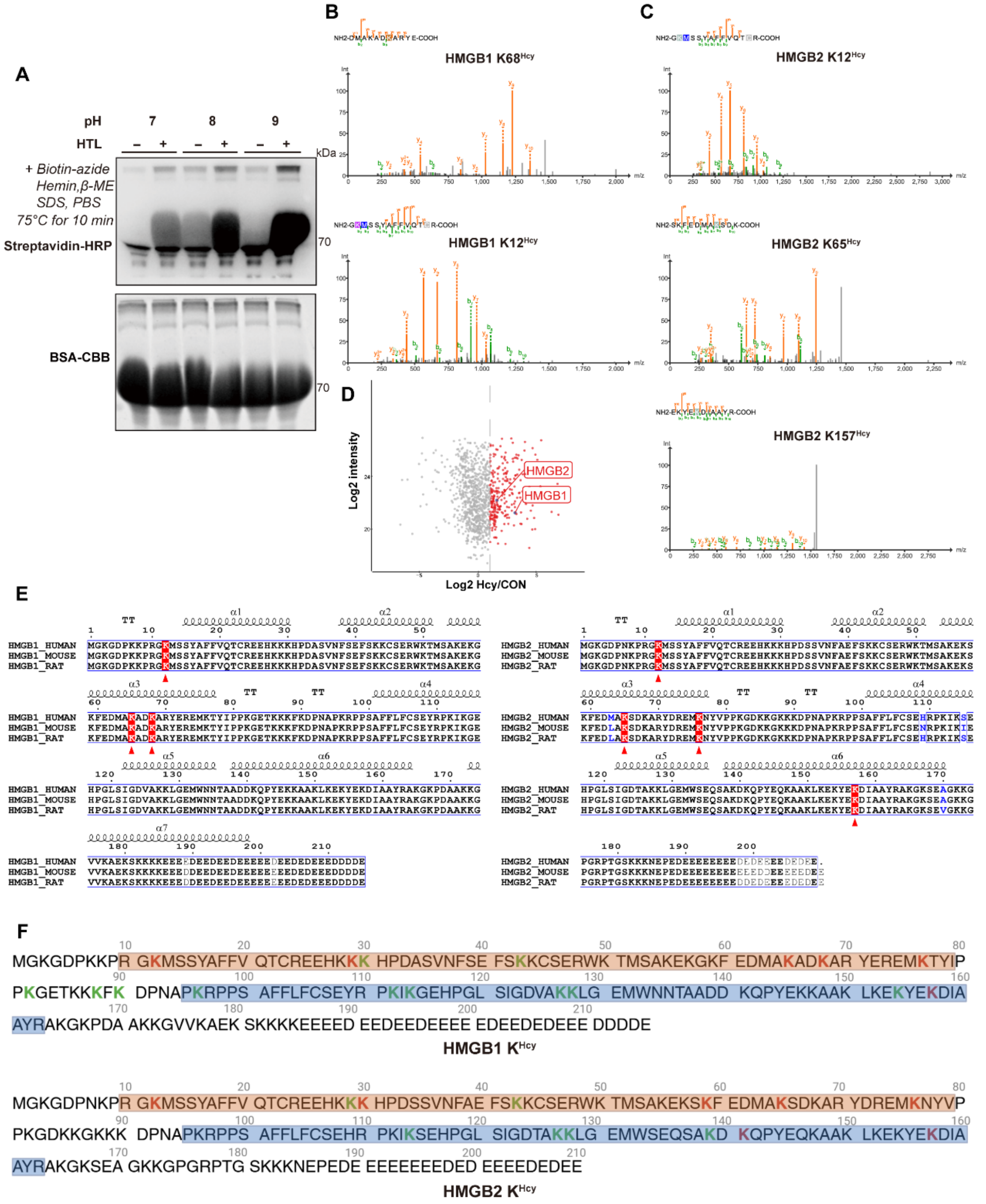

Figure S4. Hcy induces K-Hcy of HMGB1/2 in endothelial cells, related to Figure 4.

A. pH-dependent immunoblot validation of chemoselective labeling for BSA-K-Hcy under chemical treatments (biotin-azide, hemin, β-Mercaptoethanol, SDS in PBS buffer at 75°C for 10 min). Detection: streptavidin-HRP (top) with CBB loading control (bottom). **B-C.** MS/MS spectra validating K-Hcy at HMGB1 (K12/K68) and HMGB2 (K12/K65/K157) residues (ratio mod/base >0.5). **D.** Volcano plot of chemoselective

labeling-based K-Hcy differential proteomics highlighted HMGB1/2 with significant K-Hcy modification. **E.** Sequence-structural mapping of human HMGB1/2 showing K-Hcy-modified lysines (red: ratio mod/base >0.1; green: ratio mod/base ≤0.1). DNA-binding domains are annotated as Box A (red) and Box B (blue).

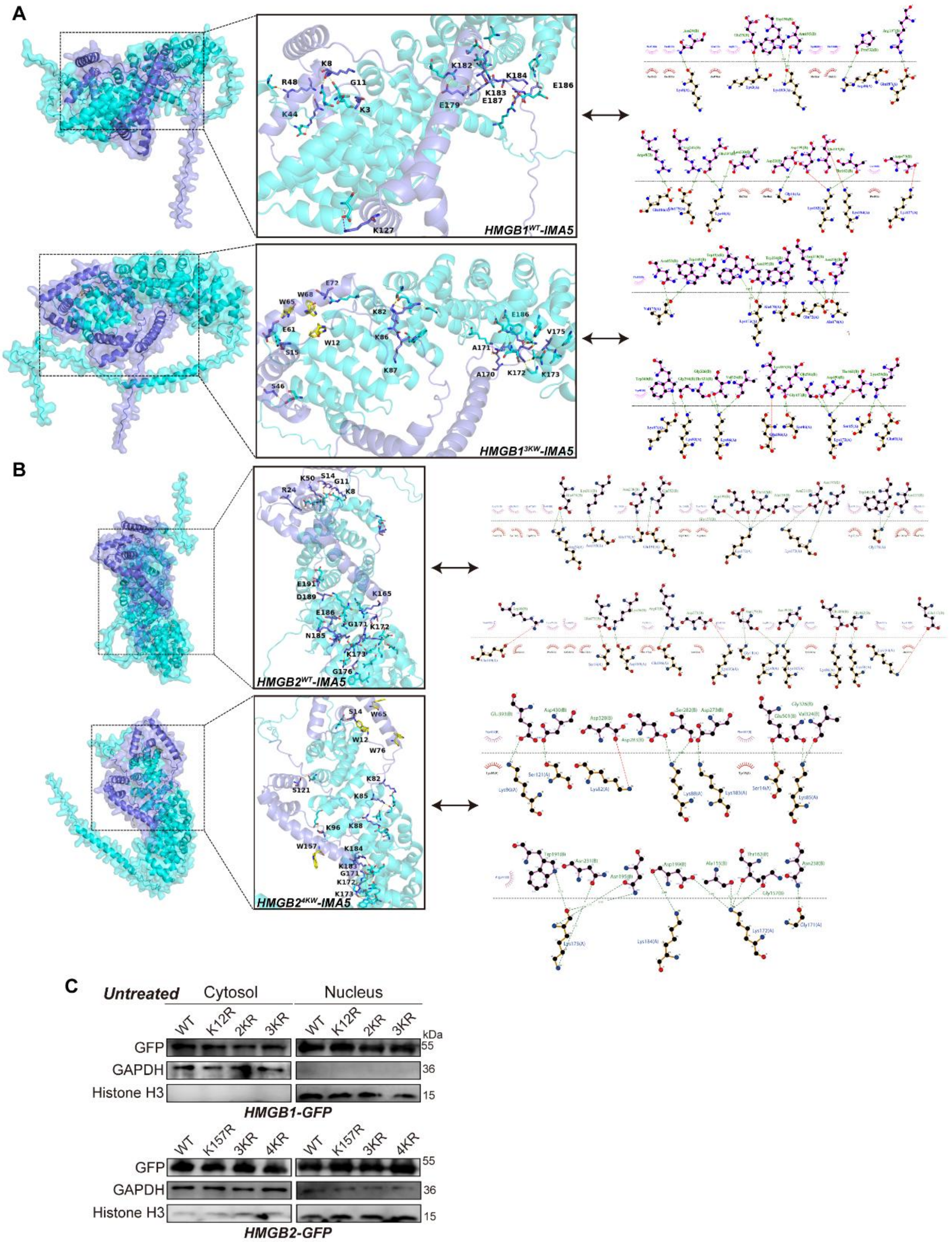

Figure S5. K-Hcy drives nuclear-to-cytoplasmic translocation of HMGB1/2, related to Figure 5.

A-B. AlphaFold3-predicted 3D structures of WT and K→W mutants of HMGB1/2 in complex with IMA5, with protein-protein interactions analyzed via PyMOL. Left panels: PyMOL-rendered complexes (HMGB1/2: violet; IMA5: cyan) with binding sites displayed as stick structures, labeled HMGB1/2 residues, and mutated residues highlighted in yellow. Right panels: Predicted interaction networks between WT or K→W mutants of HMGB1/2 and IMA5. H-bonds are shown as green dashed lines, and hydrophobic interactions as red opposite arcs. **C.** Cytosolic/nuclear fractionation immunoblots of HMGB1/2-GFP WT and lysine-to-arginine mutants in HCMECs, normalized to GAPDH (cytosol) and Histone H3 (nucleus).

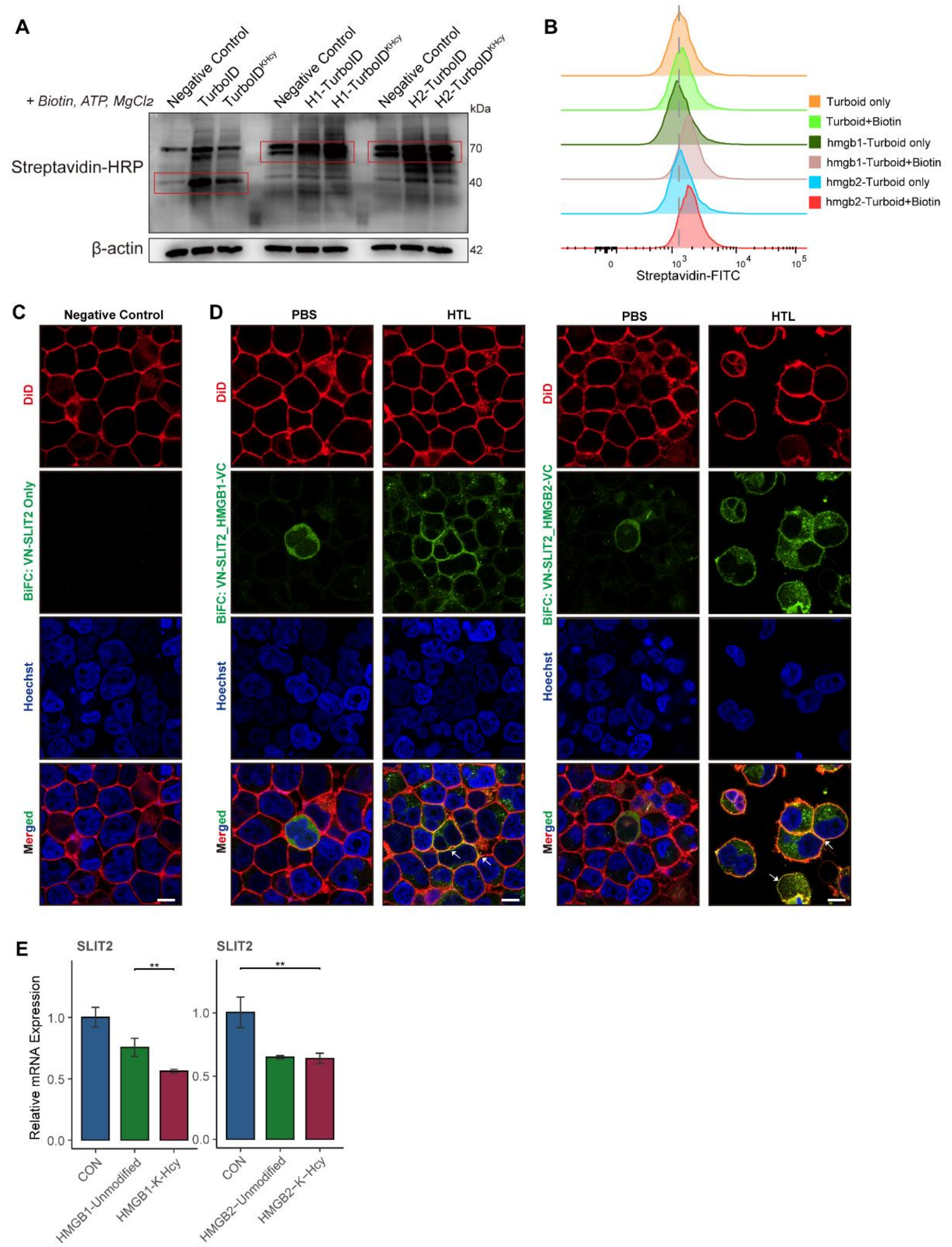

Figure S6. K-Hcy-modified extracellular HMGB1/2 enhances SASP by attenuating SLIT2-ROBO4 anti-inflammatory signaling, related to Figure 6.

A. Western blot analysis of biotinylated proteins in HCMECs treated with TurboID-only, HMGB1-TurboID (H1-TurboID), or HMGB2-TurboID (H2-TurboID) and biotin/ATP/$MgCl_2$. Red boxes highlight TurboID-dependent auto-biotinylation. **B.** Flow cytometry quantification of streptavidin-FITC fluorescence intensity in HCMECs expressing TurboID constructs ± biotin. HMGB1/2-TurboID groups exhibit biotin-

dependent signal amplification. **C.** Background BiFC signal in 293T cells transfected with VN155 (I152L)-SLIT2 alone, demonstrating negligible Venus reconstitution (green) in the absence of VC155. DiD (red): membrane staining; Hoechst (blue): nuclei. Scale bars: 10 µm. **D:** HTL (100 µM, 48h) induced significant co-localization of BiFC signals (green, VN155 (I152L)-SLIT2/HMGB1/2-VC155) with the cell membrane (DiD, red), indicating HTL-triggered extracellular release of HMGB1/2 and subsequent interaction with SLIT2. Scale bars: 10 µm. **E.** qRT-PCR analysis of *SLIT2* mRNA in HCMECs treated with unmodified or K-Hcy-modified recombinant HMGB1/2 (1 µg/mL, 24h). K-Hcy-HMGB1/2 significantly suppresses SLIT2 expression. Data normalized to ACTB (n = 3).

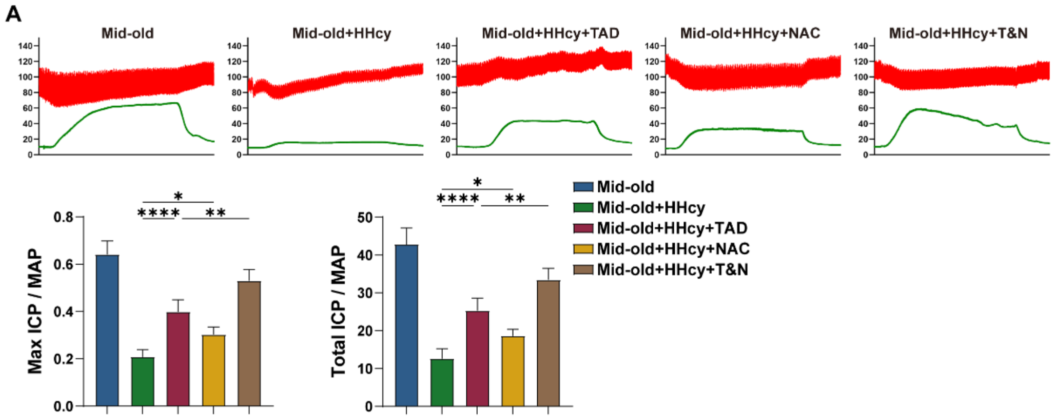

Figure S7. N-acetylcysteine attenuates endothelial senescence and rescues erectile function in middle-aged HHcy rat by blocking K-Hcy, relative to Figure 7.

A. Representative curves of electrostimulation-induced intracavernosal pressure dynamics and hemodynamic quantification (n=5 per each group). AP: arterial pressure; ICP: intracavernosal pressure; MAP: mean arterial pressure; Total ICP: area under the ICP curve.

Appendix

Table S1 Antibody used.

Antibody	Manufacture	Catalog Number	Applications
Rabbit anti HMGB1	Proteintech	10829-1-AP	WB, IF, CUT&Tag
Rabbit anti HMGB2	Abcam	ab124670	WB, IF
Rabbit anti HMGB2	Abcam	ab67282	CUT&Tag
Rabbit anti K-Hcy	This Study	N/A	WB, IP, IF
Mouse anti MARS	Abcam	ab180497	WB, IF
Mouse anti GFP tag	Proteintech	66002-1-Ig	WB, IP
Mouse anti FLAG tag	Sigma	F3165	WB, IP
Mouse anti V5 tag	Invitrogen	R960-25	WB
Rabbit anti Histone H3	Proteintech	17168-1-AP	WB
Mouse anti β-actin	Proteintech	66009-1-Ig	WB
Rabbit anti Lamin B1	Boster	BA1228	WB, IF
Rabbit anti p53	Abclonal	A0263	WB
Rabbit anti p21	Proteintech	28248-1-AP	WB, IF
Rabbit anti p21	Proteintech	10355-1-AP	WB
Mouse anti p16	Santa Cruz	sc-1661	WB
Rabbit anti Acetyllysine	PTMab	PTM-105RM	WB
Mouse anti GAPDH	Proteintech	60004-1-Ig	WB
Mouse anti GST tag	Proteintech	66001-2-Ig	WB

Table S2 Primer used.

Primer	Sequence (5'-3')	Application
MARS1 shRNA1	CCGCTGGTTTAACATTTCGTT	RNA interference
MARS1 shRNA2	TCGACATGGCAACCAATATAT	RNA interference
MARS1-Forward	AGGCCCGGAAGATTGTGTG	qRT-PCR
MARS1-Rerverse	AGAGGTAGTTGCCGCTATCCA	qRT-PCR
TNF-Forward	GAGGCCAAGCCCTGGTATG	qRT-PCR
TNF-Rerverse	CGGGCCGATTGATCTCAGC	qRT-PCR
IL1B-Forward	AGCTACGAATCTCCGACCAC	qRT-PCR
IL1B-Rerverse	CGTTATCCCATGTGTCGAAGAA	qRT-PCR
MMP3-Forward	AGTCTTCCAATCCTACTGTTGCT	qRT-PCR
MMP3-Rerverse	TCCCCGTCACCTCCAATCC	qRT-PCR
MMP13-Forward	ACTGAGAGGCTCCGAGAAATG	qRT-PCR
MMP13-Rerverse	GAACCCCGCATCTTGGCTT	qRT-PCR
IL6-Forward	ACTCACCTCTTCAGAACGAATTG	qRT-PCR

IL6-Rerverse	CCATCTTTGGAAGGTTCAGGTTG	qRT-PCR
SERPINE1-Forward	ACCGCAACGTGGTTTTCTCA	qRT-PCR
SERPINE1-Rerverse	TTGAATCCCATAGCTGCTTGAAT	qRT-PCR
SLIT2-Forward	AGCTTAGACGAATTGACCTGAGC	qRT-PCR
SLIT2-Rerverse	CCGAAGGCAGTTTATCTTGTTGG	qRT-PCR
ACTB-Forward	GTCATTCCAAATATGAGATGCGT	qRT-PCR
ACTB-Rerverse	GCTATCACCTCCCCTGTGTG	qRT-PCR

Table S3 Recombinant DNAs used.

Recombinant DNAs	Application
pcDNA3.1-tPA-SP-Flag-HMGB1-TurboID<br>pcDNA3.1-tPA-SP-Flag-HMGB2-TurboID<br>pcDNA3.1-tPA-SP-Flag-TurboID	TurboID Proximity Labeling
pLenti-EnCMV-HMGB1-EGFP-Puro<br>pLenti-EnCMV-HMGB1 (K12W/R)-EGFP-Puro<br>pLenti-EnCMV-HMGB1 (K65, 68W/R)-EGFP-Puro<br>pLenti-EnCMV-HMGB1 (K12, 65, 68W/R)-EGFP-Puro<br>pLenti-EnCMV-HMGB2-EGFP-Puro<br>pLenti-EnCMV-HMGB2 (K157W/R)-EGFP-Puro<br>pLenti-EnCMV-HMGB2 (K12, 65, 76W/R)-EGFP-Puro<br>pLenti-EnCMV-HMGB2 (K12, 65, 76, 157W/R)-EGFP-Puro	Nucleocytoplasmic Distribution Assay
pBiFC-VN155 (I152L)-SLIT2-N<br>pBiFC-VC155-ROBO4<br>pBiFC-HMGB1-VC155<br>pBiFC-HMGB2-VC155	BiFC
pLKO.1-U6-MARS1-shRNA1<br>pLKO.1-U6-MARS1-shRNA2<br>pLenti-EnCMV-MARS1-Myc-Puro	Bidirectional Gene Modulation
pLenti-EnCMV-HMGB1-Flag-Puro<br>pLenti-EnCMV-HMGB2-Flag-Puro	IP
pGEX-4T-1-HMGB1<br>pGEX-4T-1-HMGB2<br>pcDNA3.1-SLIT2-V5<br>pcDNA3.1-SLIT2-N (31~1121 aa)-V5<br>pcDNA3.1-SP (1~30 aa)-SLIT2-C (1122~1529 aa)-V5	GST pull-down
